# Sustainable improvement of interprofessional care for better resident outcomes: protocol for the INTERSCALE hybrid type III effectiveness cluster-randomized trial comparing individualized and collaborative delivery of an evidence-based care model for long-term care

**DOI:** 10.1186/s13012-026-01489-0

**Published:** 2026-02-20

**Authors:** Franziska Zúñiga, Lea Saringer-Hamiti, Flaka Siqeca, Sarah Holzer, Raphaëlle-Ashley Guerbaai, Thekla Brunkert, Farah Islam, Jana Bartáková, Anja Orschulko, Sandra Staudacher, Reto W. Kressig, Andreas Zeller, Christine Serdaly, Nathalie I. H. Wellens, Sabina M. De Geest, Vanessa Litschgi, Natalie Zimmermann, Michael Simon

**Affiliations:** 1 Nursing Science, Department of Public Health, University of Basel, Basel, Switzerland; 2 Academic Centre for Nursing and Midwifery, Department of Public Health and Primary Care, KU Leuven, Leuven, Belgium; 3 Rehabilitation, Ageing, and Independent Living (RAIL) Research Centre, School of Primary and Allied Health Care, Monash University, Frankston, Australia; 4 Center for Primary and Community Care, Faculty of Health Sciences and Medicine, University of Lucerne, Lucerne, Switzerland; 5 Centre for Primary Health Care, University of Basel, Basel, Switzerland; 6 Serdaly&Ankers snc, Conches, Switzerland; 7 La Source School of Nursing, HES-SO University of Applied Sciences and Arts of Western Switzerland, Av. Vinet 30, Lausanne, Switzerland; 8 Medical Faculty, University of Basel, Basel, Switzerland; 9 Health Economics Facility, Department of Public Health, University of Basel, Basel, Switzerland

**Keywords:** Long-term care, Implementation strategies, Care model, Cluster randomized trial, Implementation Science, Unplanned hospital transfers

## Abstract

**Background:**

Over recent decades, multifaceted nurse-led care models have been developed to reduce unplanned hospital transfers from long-term care facilities (LTCFs). In Switzerland, the INTERCARE model has demonstrated effectiveness, with core components including deployment of nurses in expanded roles (INTERCARE nurses), evidence-based communication tools, and advance care planning. However, resource-intensive implementation strategies such as 1:1 support meetings for model implementers pose challenges for scale-up, underscoring the need for more scalable implementation support. The INTERSCALE study compares two modes of delivering implementation support—an individualized and a collective-oriented approach—testing the hypothesis that the latter achieves non-inferior fidelity to the INTERCARE model and comparable reductions in unplanned hospital transfers at the LTCF level. Secondary aims are to compare implementation (acceptability, feasibility), economic (costs, cost-effectiveness), clinical (unplanned transfers), and organizational (staff absences, turnover) outcomes.

**Methods:**

This non-inferiority, effectiveness–implementation hybrid type III trial uses a cluster-randomized controlled design, with LTCFs as the unit of randomization. Forty German-speaking LTCFs in Switzerland (≥20 long-term care beds; cantonal accreditation) will be randomized (1:1) after formal consent to either individualized or collective implementation support, without blinding of LTCFs or the research team. In the individualized arm (20 LTCFs), leadership receives 1:1 support meetings, and INTERCARE nurses receive 1:1 coaching, mirroring the original INTERCARE trial. In the collective arm (20 LTCFs), leadership support and INTERCARE nurse coaching are delivered in group formats involving several LTCFs/INTERCARE nurses together at two-monthly intervals. The primary outcome is LTCF-level fidelity to the INTERCARE core components, analyzed with a binomial generalized linear mixed model including a random LTCF effect. Non-inferiority of the collective mode will be concluded if the lower bound of its 95% confidence interval for fidelity is within 15% of the individualized mode. A 12-month cost-effectiveness analysis from a multi-stakeholder perspective (LTCFs and research group) will estimate the incremental cost-effectiveness ratio using differences in implementation costs and unplanned transfers between arms; secondary outcomes include unplanned transfers, staff turnover, and absences.

**Discussion:**

This type III hybrid cluster trial addresses a key scaling challenge in implementation science by testing less resource-intensive implementation strategies for disseminating an evidence-based care model across LTCFs in routine practice.

**Trial registration:**

Prospectively registered on June 25, 2024, at ClinicalTrials.gov nr. NCT06473051.

**Supplementary Information:**

The online version contains supplementary material available at 10.1186/s13012-026-01489-0.

Contributions to literature

- INTERSCALE evaluates the scale-up of the nurse-led INTERCARE model, previously shown to reduce unplanned hospital transfers from long-term care facilities (LTCFs), by comparing two modes of delivering implementation support to LTCF leadership and nurses in expanded roles: individualized one-to-one support versus collaborative group-based support across multiple LTCFs.
- This study will deepen understanding of which implementation support strategies are effective for implementing and scaling evidence-based care models in LTCFs, with a focus on feasibility in routine practice.
- INTERSCALE systematically compares the costs and effects of two modes of delivering the same implementation strategies, enabling an assessment of the model’s cost-effectiveness for reducing unplanned hospital transfers at the organizational (LTCF) level.

## Background

Over the last two decades, nurse-led care models have been developed to reduce unplanned hospital transfers, achieving a reduction of 6.1% to 11.7% in unplanned transfers and 30% in overall transfers [1–3]. Given that between 19% and 67% of unplanned hospital transfers from long-term care facilities (LTCFs) may be avoidable) [4–7] and that such transfers increase residents’ risk of adverse events and overall health care costs [8, 9], nurse-led care models offer a promising approach to improving system-level care for older adults. These models typically position nurses in expanded roles as clinical leaders who coach and empower teams and support interdisciplinary decision-making regarding transfers. In multidisciplinary teams, these nurses may be advanced practice nurses (e.g., clinical nurse specialists) or registered nurses (RNs) with additional training, responsible for comprehensive geriatric assessments, root-cause analyses, benchmarking of hospital transfers and quality indicators, and advance care planning with residents and relatives [10].

Despite their promise, few evidence-based models that reduce unplanned transfers have been successfully scaled up. The EVERCARE model, which paired primary care physicians with nurse practitioners to reduce preventable hospital admissions in US LTCFs, proved less effective when implemented in the UK, partly due to poor information systems [11, 12], underscoring the importance of a careful contextual analysis before scale-up. Similarly, the MOQI model, which reduced hospitalizations in Missouri LTCFs by 30% [13], was successful in some facilities but not in others, possibly due to insufficient leadership engagement and support for local change [12]. Overall, there are limited examples of healthcare interventions that have been successfully taken to scale [14].

In Switzerland, the INTERCARE nurse-led care model was developed and demonstrated effectiveness in reducing unplanned hospital transfers in 11 LTCFs [15]. INTERCARE comprises six core components: 1) an INTERCARE nurse, a registered nurse with geriatric expertise in an expanded role who also acts as an implementer to introduce other components; 2) strengthened interprofessional collaboration; 3) advance care planning; 4) evidence-based communication tools (STOP&WATCH, ISBAR); 5) comprehensive geriatric assessment; and 6) data-driven quality improvement. Designed to drive organizational change, the model equips LTCF leadership and nurses in expanded roles with new skills and supports them to introduce and sustain new processes. In addition to reducing unplanned transfers, anecdotal evidence in the original study suggested possible organizational effects on staff turnover and absences. However, the impact of nurses in expanded roles on these outcomes remains largely unexplored [16]. INTERCARE showed high acceptability, fidelity, and sustainability, but required substantial time and financial investment in personnel resources from the research team to provide implementation support [15, 17–19].

### Implementation strategies of nurse-led models

LTCFs are complex organizations in which implementing innovations is challenging due to multilevel influences, continuous change, high staff turnover, resource constraints, top-down management, and communication problems [20]. To address these challenges, locally tailored and cost-effective implementation strategies are pivotal, where implementation strategies are understood as techniques that enhance the adoption, implementation, and sustainability of evidence-based interventions such as INTERCARE [21].

Existing studies on nurse-led care models in LTCFs report varied implementation strategies targeting both staff and leadership. From the perspective of the Interactive Systems Framework for Dissemination and Implementation (ISF), these strategies primarily focus on the delivery system level [22]. The ISF distinguishes three systems: the delivery system, which implements the innovation; the support system, which supports the delivery system in implementation; and the synthesis and translation system, which synthesizes research and prepares it for use by the other two systems. Common strategies to support LTCF staff include training, protocols, clinical benchmarking, champions, and new clinical teams. In contrast, leadership-level strategies are less frequent and mainly consist of clinical benchmarking and training (e.g., in quality improvement methods and good practices) [11, 23–29]. These strategies are often delivered by research teams or private companies, representing the ISF’s support system level. However, the most effective strategies for implementation and sustainment remain unclear, are highly context-dependent, and are often poorly distinguished, inconsistently reported, and entangled with intervention descriptions, hampering cross-study comparisons [30, 31]. Economic analyses are lacking, and few studies report on strategies during the sustainment phase.

During the INTERCARE study, LTCFs greatly valued the implementation support provided by the research team, particularly the bimonthly support meetings with leadership and the biweekly one-to-one coaching sessions for INTERCARE nurses—strategies rarely described in the literature. These meetings enabled teams to reflect on progress, address barriers, and co-develop solutions, and were perceived as key to successful implementation. However, because individualized meetings and coaching sessions were the most resource-intensive strategies for the research team, they limit scalability to additional LTCFs and, by extension, the model’s broader population impact. Therefore, more cost-efficient modes of delivering implementation support need to be examined, such as collaborative support meetings across multiple LTCFs and group-based coaching for INTERCARE nurses.

There is substantial evidence that group learning enables individuals to learn more effectively by engaging with peers and exchanging diverse perspectives. Social Cognitive Learning Theory (SCLT) posits that people learn by observing others’ actions and considering the consequences of those actions [32]. Because learning occurs through observation, modelling, and social interaction, SCLT emphasizes that learning often happens naturally in group settings where individuals can watch, imitate, and receive feedback from others [32]. Evidence from systematic reviews indicates that group and collaborative processes are critical facilitators of innovation implementation and improved outcomes in healthcare settings: McGuier et al. [33] identified effective communication and shared cognitive states as fundamental mechanisms through which teams collectively learn, coordinate, and implement new practices. Another review of implementation strategies in nursing practice showed that formal group learning methods and strategies leveraging social influence (e.g., opinion leaders) are statistically effective in changing practice [34]. Together, these findings suggest that collaborative learning approaches may strengthen implementation success.

To evaluate and compare implementation strategies, fidelity is a commonly used outcome, defined as the extent to which an intervention or procedure is delivered as intended [35]. Strategies that support effective implementation are expected to enhance adherence to core components; higher fidelity allows an intervention to exert its intended effect and is often associated with improved health outcomes [36]. Fidelity and sustainability – defined as the continued use of core components after implementation – are therefore key outcomes when comparing implementation strategies or, in this case, different modes of delivering implementation strategies when preparing a complex intervention for full-scale implementation.

### Scaling-up effective interventions

Scale-up has been defined as replicating and extending the reach of an intervention into other localities, cities, or regions [37], with the aim of achieving sustainable health benefits [37, 38]. Scale-up can be positioned at the pragmatic end of the explanatory-pragmatic continuum, focusing on moving real-world evidence into community and system-level practice [39]. Scaling up effective interventions enables additional settings and populations to benefit from practices proven successful in controlled research conditions and supports sustainable policy and program development [40].

However, several pitfalls in preparing for scale-up have been reported, including a lack of resources and contextual barriers, such as difficulties in adapting interventions to new settings [41]. Limited reporting and poor specification of implementation methods and strategies have further led to insufficient evidence to support scale-up, particularly when interventions and their implementation strategies are not described in sufficient detail to enable uptake and replication by government agencies, health care organizations, or foundations [42, 43]. This underscores the need for rigorously designed studies that both test scale-up and explicitly specify implementation strategies and their delivery.

**Fig. 1:**
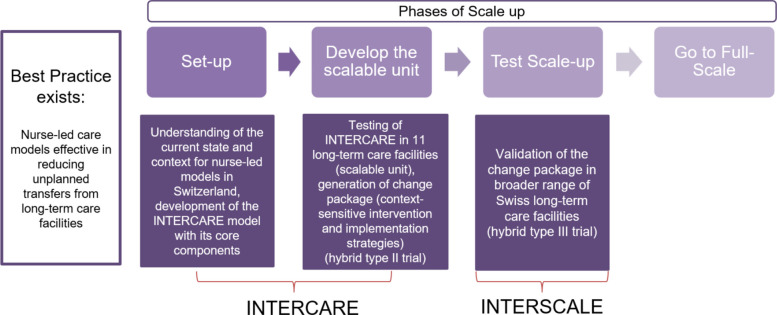
Different phases to reach full-scale implementation, adapted based on Barker and colleagues (2016) [44]

Figure 1 illustrates how INTERSCALE fits into Barker and colleagues’ framework for scaling up health interventions [44], moving from an existing best-practice model to full scale. The process began with 1) building the INTERCARE nurse-led model based on existing international evidence and close engagement with early adopters and local stakeholders (set-up), and 2) testing the model in a first scalable unit of 11 LTCFs in the German-speaking part of Switzerland and developing a change package that includes both the context-sensitive complex intervention with six core components and the corresponding implementation strategies (develop scalable unit). It will continue with 3) validating INTERCARE in the INTERSCALE study in a test scale-up while evaluating cost-effective implementation strategies (test scale-up), and will eventually lead to 4) preparing for full-scale implementation [44].

### The INTERSCALE study

**Fig. 2:**
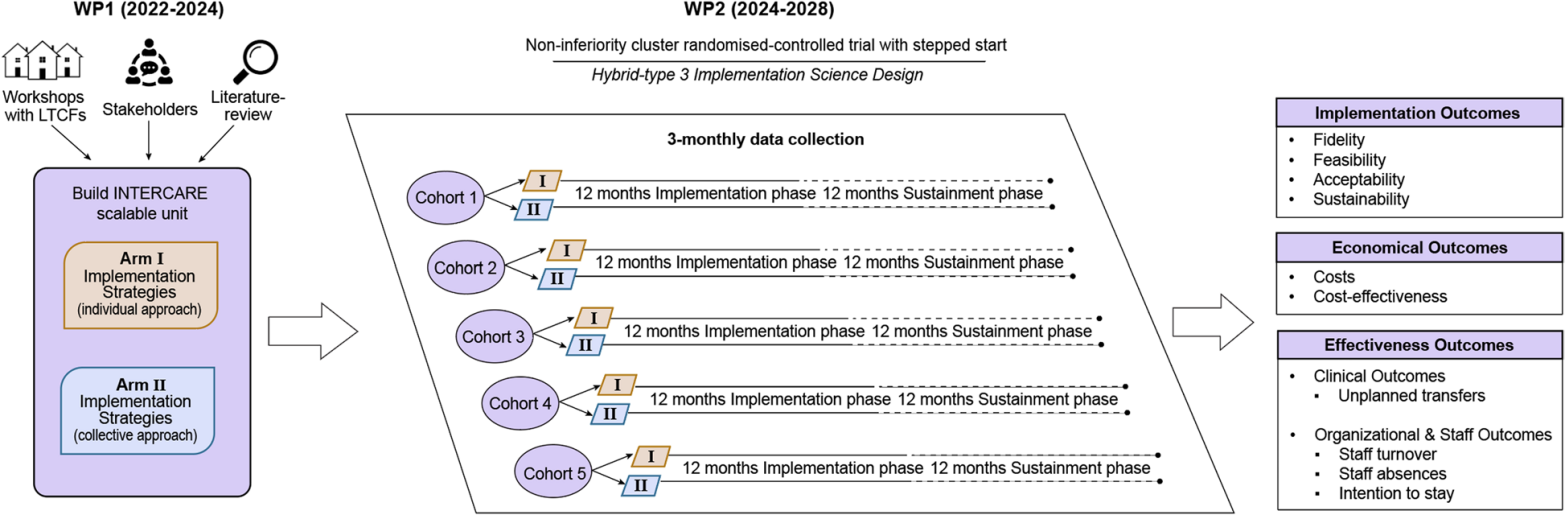
The INTERSCALE study

The INTERSCALE study (Sustainable improvement of INTERprofessional care for better resident outcomes - SCAling up an Evidence-based care model for nursing homes) has two phases, each corresponding to a work package (WP). In WP1, the change package (i.e., the context-sensitive core components and corresponding implementation strategies) was developed, building on the existing material and insights from INTERCARE, which will then be tested in a second work package (WP2) (see Fig. 2). INTERSCALE thus constitutes a test scale-up of the INTERCARE model while simultaneously generating evidence on a more cost-effective mode of delivering implementation strategies (individualized vs. collaborative support meetings and coaching sessions at both LTCF leadership and INTERCARE nurse levels). This approach is intended to build toward full-scale implementation and address previously identified scalability challenges of INTERCARE for LTCFs.

The central hypothesis is that replacing individual LTCF support with group-based support, and one-to-one INTERCARE nurse coaching with collaborative coaching, will reduce costs by minimizing the time investment of the support system (research team and external facilitators), while maintaining fidelity to the care model at LTCF level and preserving its clinical effect in reducing residents’ unplanned transfers.

The Exploration, Preparation, Implementation, Sustainment (EPIS) framework guides INTERSCALE [45]. WP1 corresponds to the preparation phase, in which minimal requirements of the core components were adapted and implementation strategies – along with the two delivery modes to be compared – were defined; WP2 covers the LTCFs’ preparatory, implementation, and sustainment phases.

Several methodological considerations informed the development of implementation strategies for WP2. Four workshops were conducted with LTCFs that had participated in INTERCARE and those interested in INTERSCALE. Using an implementation mapping approach [46], strategies were adapted to enhance adoption, implementation, and sustainability of the INTERCARE model. The minimal requirements for each core component were reviewed to focus on essential functions and distinguish these from the various ways components may be operationalized in practice [47], followed by adaptations using the FRAME method to align the model and strategies with the Swiss context [48]. In the first workshop, LTCFs identified barriers and facilitators and suggested adaptations. Performance objectives were then defined to address these barriers (see Supplementary Material 1), and the COM-B model and the Theoretical Domains Framework (TDF) were applied to identify behavioral determinants [49]. Finally, implementation strategies were adapted based on these determinants and described using the action, actor, context, target, time (AACTT) framework of Presseau and colleagues [50], resulting in a set of clearly specified strategies to be examined in the planned cluster-RCT (see Supplementary Material 2).

### Development of implementation strategies for INTERSCALE

For the planned cluster-RCT in WP2, two alternative modes of delivering implementation strategies were considered. To improve cost-effectiveness, the team initially explored removing strategies from INTERCARE or replacing them with less costly alternatives; however, workshop feedback indicated that LTCFs perceived all existing strategies as appropriate and necessary for successful implementation. Given that coaching of INTERCARE nurses and onsite support meetings with LTCF model implementers (i.e., local professionals responsible for implementing the model, including INTERCARE nurses) were the costliest strategies, shifting from individualized LTCF support and one-to-one coaching to collaborative formats was identified as the most feasible cost-saving approach. Limited expertise in external facilitation for long-term care, especially in organizational development, further supports concentrating expert resources in group sessions.

**Fig. 3:**
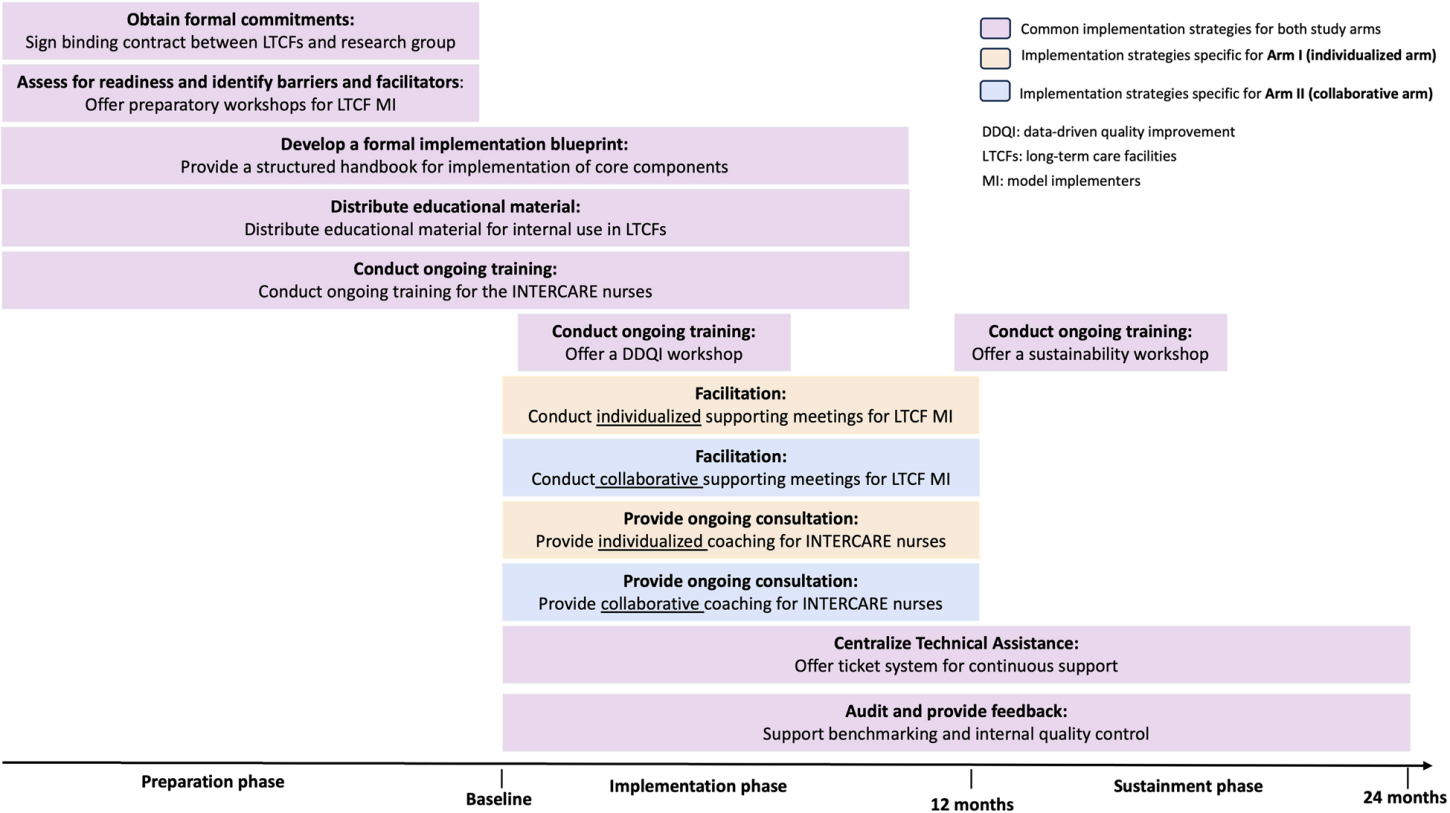
Implementation strategies per EPIS phase in work package 2

In addition, based on the WP1 workshops, two strategies were added to both study arms: 1) an implementation handbook describing INTERCARE’s core components, minimal requirements, performance objectives, barriers and facilitators, frequently asked questions, and resource materials; and 2) a workshop on data-driven quality improvement, as this core component had been poorly understood in the original study. A ticketing system was also introduced to streamline questions arising between support meetings by channeling email requests into a structured system, thereby changing the mode of delivery for continuous support compared with personal emails in INTERCARE. Figure 3 summarizes all implementation strategies across three phases of the EPIS framework, highlighting differences between the two arms in two strategies: facilitation and ongoing consultation.

This protocol focuses on WP2, which includes the LTCFs’ preparatory, implementation, and sustainment phases, and will test the INTERCARE change package developed in WP1 to determine whether a shift from individualized to collaborative support can achieve similar implementation and clinical outcomes [15].

### Aims

The overall aim of INTERSCALE is to compare two modes of delivery for support and coaching (individualized vs. collaborative) to help LTCFs adopt and implement the INTERCARE model. This includes assessing implementation outcomes, calculating cost-effectiveness, and evaluating overall effectiveness. Specifically, the aims are:

- Primary outcome: To compare the individualized vs. collaborative arm in relation to LTCFs’ overall fidelity to the INTERCARE model (aim 1).
- Secondary implementation outcomes: To compare the two modes of delivery in relation to further implementation outcomes, i.e., LTCFs’ fidelity to the six single core components of the INTERCARE model, sustainability of the INTERCARE model, as well as the model’s acceptability and feasibility (secondary aim 2.1).
- Economic outcome: to assess the costs of the implementation strategies and to calculate the incremental cost-effectiveness ratio (ICER) of implementing the INTERCARE model in LTCFs. This will involve comparing the two modes of delivery from both the LTCFs' and the research team's perspectives, with the number of unplanned hospital transfers of LTC residents as the effectiveness outcome (secondary aim 2.2).
- Effectiveness outcomes:
  o To assess the effect of the two modes of delivery on clinical effectiveness, specifically unplanned transfers of LTCF residents (secondary aim 2.3a).
  o To assess the effect of the two modes of delivery on organizational outcomes, such as staff absences and staff turnover (aim 2.3b).

## Methods

### Study design

**Fig. 4:**
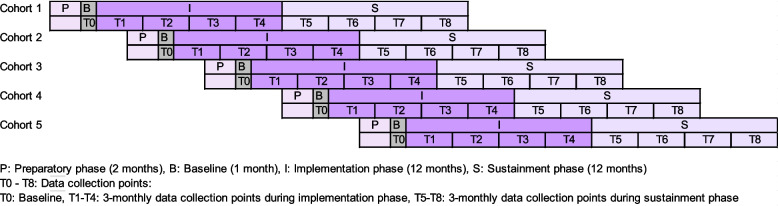
Design for cluster-randomized controlled trial and data collection points

The trial follows CONSORT guidelines for cluster-randomized controlled trials (RCT) (Supplementary Material 3) [51]. This is a non-inferiority effectiveness–implementation hybrid type III cluster-RCT [52, 53], with LTCFs as the unit of randomization to reflect that the intervention targets organizational routines rather than individual residents (Fig. 4).

An explanatory mixed-methods process evaluation focusing on implementation strategies will accompany the trial but is not part of this protocol.

### Setting

The study takes place in the German-speaking part of Switzerland. In 2023, the Swiss Federal Office of Statistics reported 1,480 LTCFs with 100,727 beds, of which 1,117 LTCFs are in the German-speaking part [54]. Registered nurses account for about 30% of the skill mix in Swiss LTCFs, with most medical care provided by general practitioners (GPs); around 45% of LTCFs in the German-speaking part of Switzerland have a contract with a single physician, while about 90% work with several GPs (mean 16) [55, 56].

### Sample size and inclusion criteria

**Fig. 5:**
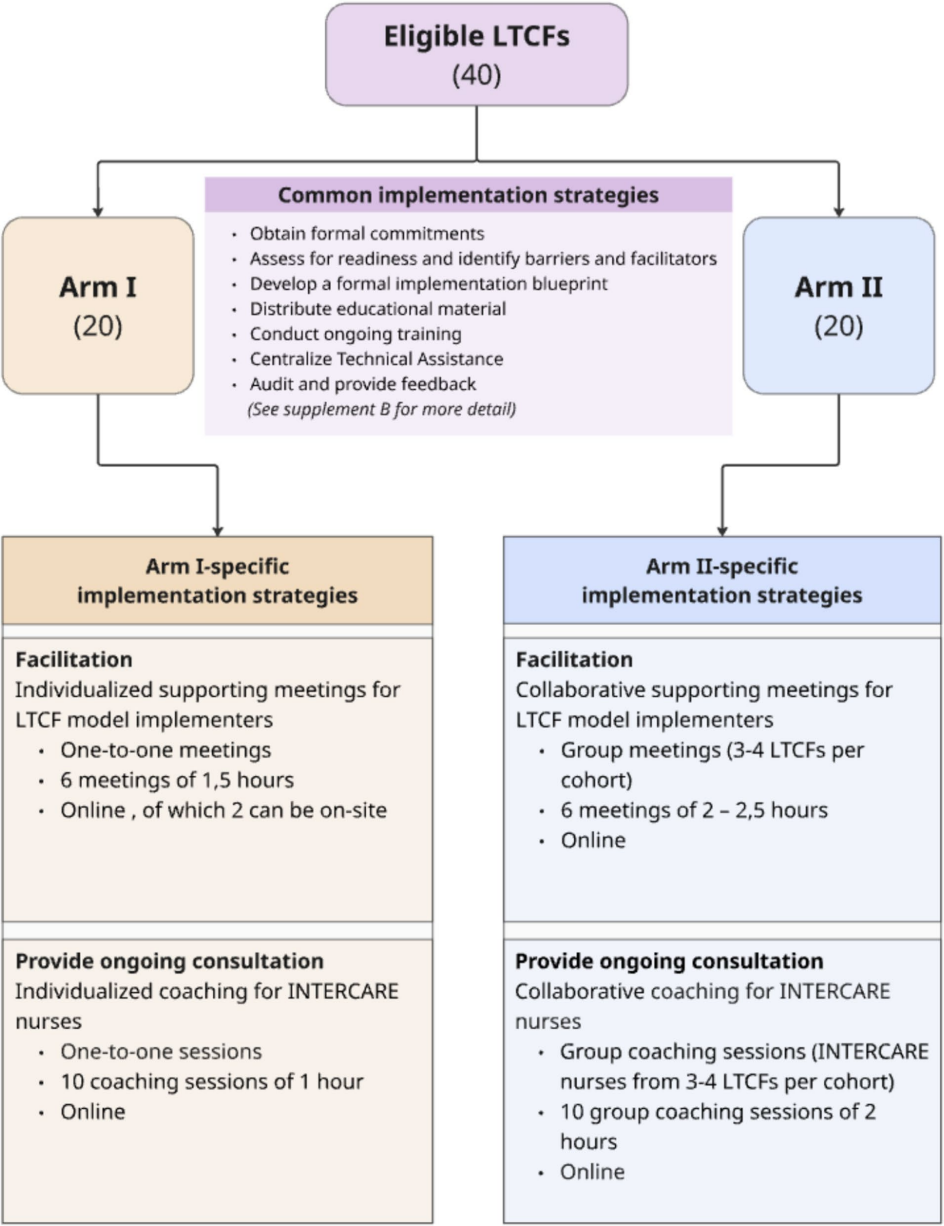
Flow diagram for planned recruitment and differentiation of study arms

LTCFs will be recruited via LTC national and professional associations, magazines, direct contact, and snowballing. The sample size calculation assumes a 15% difference in overall fidelity between trial arms (65% vs. 80%). Based on a simulation with 2,000 iterations, a between-cluster variance of 0.1 (ICC = 0.029), and a two-sided alpha of 0.025, 18 LTCFs per arm provide 84% power to detect a 15% difference; for a 20% difference, power increases to 97%. Allowing for a 10% loss between consent and allocation, and after intervention start, the target sample is 40 LTCFs (20 per arm) (Fig. 5).

Inclusion criteria at the cluster level (LTCF) and within clusters are described in Table 1. Within each cluster, all eligible participants (INTERCARE nurses, residents, staff, physicians, and LTCF leadership) who meet the inclusion criteria will be included.

**Table 1:** Inclusion and exclusion criteria

**Staff** **(expected number of participants)**	**Inclusion criteria**	**Exclusion criteria**
LTCFs (cluster level)	• German-speaking • ≥20 long-term care beds • Cantonal accreditation • Formal commitment to introduce the INTERCARE core components • Willingness to provide routine resident assessment data	• Already working with INTERCARE nurses and existing high fidelity to INTERCARE core elements
INTERCARE nurse (1–3 per LTCF)	• Employment or formal affiliation with the LTCF • At least a registered nurse degree and three years’ experience working with older adults • Formal assignment as an INTERCARE nurse	• Temporary substitute of INTERCARE nurse (e.g., during holidays)
Residents (ca. 4,000 in total)	• All residents present during the study period who are billed under the health insurance law	• Opt-out by residents or their legal representative • Short-stay residents
Care staff (ca. 3,200 in total)	• All direct care staff (e.g., care workers, physiotherapists, activity staff) at different educational levels (RNs, licensed practical nurses, nurse aides) who work ≥8 hours/week for ≥3 months • Therapeutic personnel involved in treatments ≥8 hours/week	• Non-healthcare professionals (e.g., administrative staff)
General Practitioner (GP) (ca. 400 in total)	• Treating at least five residents in the LTCF at the time of the survey	• Temporary substitute GPs
LTCF leadership (ca. 80 in total)	• LTCF director, director of nursing, project manager (if different), relevant unit leaders, and other designated personnel (e.g., quality manager, needs assessment instrument supervisors)	• Leadership person not involved in the study

### Randomization

Allocation of LTCFs to each arm will be concealed from recruiters in the project team. A project collaborator not involved in recruitment will use computer-generated random numbers to allocate LTCFs (simple randomization) after LTCFs have signed the formal commitment and consented to participate [57]. Allocation will remain concealed from LTCFs, staff, and the research team until all LTCFs in the cohort are assigned; thereafter, both researchers and LTCFs will be aware of the allocation. The timing of cohort assignment and implementation start will be discussed with LTCFs based on their self-reported readiness to implement and their appointment of an INTERCARE nurse.

### Intervention

The intervention is the INTERCARE nurse-led care model with six core components (INTERCARE nurse, interprofessional collaboration, advance care planning, evidence-based communication tools, comprehensive geriatric assessment, and data-driven quality improvement), as detailed in Supplement 1 and the previous INTERCARE protocol [58]. For each core component, minimal requirements – deemed essential for effectiveness – must be introduced (e.g., ACP conversation with each newly admitted resident). These minimal requirements were refined in WP1 to align with updated evidence and context.

To support implementation, performance objectives are defined for each minimal requirement (e.g., the INTERCARE nurse, together with their supervisors and the unit leaders, develops a work plan that outlines designated time slots for regular exchanges with each unit). Peripheral elements beyond minimal requirements describe acceptable variants (e.g., job-sharing for INTERCARE nurses), which LTCFs may adopt as appropriate.

### Implementation strategies, mode of delivery, and study arms

In WP1, implementation strategies were specified and differentiated between the two study arms (Fig. 3). Strategies target LTCF model implementers, LTCF leadership (e.g., LTCF director, director of nursing), and INTERCARE nurses and are fully described using the AACTT framework [50] in Supplementary Material 2.

The key contrast between arms is the mode of delivery of support meetings for LTCF model implementers and coaching for INTERCARE nurses. In arm I (individualized support), support meetings with LTCF model implementers and coaching sessions with INTERCARE nurses are held separately for each LTCF and for each INTERCARE nurse, respectively. In arm II (collaborative support), LTCF model implementers and INTERCARE nurses are grouped across LTCFs within the same cohort arm for joint support and coaching sessions.

### Data collection

Data are collected at baseline and then every three months during the implementation and sustainment phases (Fig. 4). Data sources include online surveys, structured reports, semi-structured interviews, observations, Excel data sheets, and the extraction of routine data and staff rosters for implementation, economic, and effectiveness outcomes (Table 2).

**Table 2:** Data collection points in view of study aims and target population

				**Intervention phase**				**Sustainment phase**			
	**Target population**	**Data source**^**a**^	**T0**	**T1**	**T2**	**T3**	**T4**	**T5**	**T6**	**T7**	**T8**
**Implementation outcome**											
Overall fidelity score (intervention phase) (aim 1)	LTCF leadership	Semi-structured interview (quan. and qual. data)	x	x	x	x	x				
Fidelity to core components (aim 2.1):											
a) Interprofessional collaboration	INTERCARE nurse, LTCF staff	Online survey	x		x		x				
	Physicians				x		x				
b) INTERCARE nurse	INTERCARE nurse	Structure report about coaching activities (qual. data)		x	x	x	x	x	x	x	x
		Observations (qual. data)			x						
c) Comprehensive Geriatric Assessment	LTCF leadership	Structure report about activities related to CGA (qual. data)					x				
d) Evidence-based tools	LTCF staff	Online survey	x		x		x				
	Physicians				x		x				
e) Advance Care Planning	Residents (reported by LTCF leadership/INTERCARE nurse)	Excel data sheet	x	x	x	x	x	x	x	x	x
f) Data-driven quality improvement	LTCF leadership/project team	Structured report about activities related to data-driven quality improvement (qual. data)					x				
Sustainability (aim 2.1)	LTCF leadership	Semi-structured interview (quan. and qual. data)						x	x	x	x
Acceptability (aim 2.1)	LTCF leadership (only T0 and T4), INTERCARE nurse, LTCF staff	Online survey	x		x		x				
Feasibility (aim 2.1)	LTCF leadership (only T0 and T4), INTERCARE nurse, LTCF staff	Online survey	x		x		x				
**Economic outcomes**											
Costs of implementation strategies (aim 2.2)	LTCF leadership, staff involved in implementing INTERCARE; INTERSCALE research team	Online form	x	x	x	x	x	x	x	x	x
Wages, price list and transfer’s impact on LTCF revenues (aim 2.2)	LTCF leadership	Questionnaire		x				x			x
**Effectiveness outcomes**											
Unplanned transfers (aim 2.3a)	Residents (reported by LTCF leadership/INTERCARE nurse)	Excel-based quality monitoring tool	x	x	x	x	x	x	x	x	x
Staff turnover (aim 2.3b)	LTCF leadership	LTCF routine data/exports from staff rosters	x	x	x	x	x	x	x	x	x
Staff absences (aim 2.3b)	LTCF leadership	LTCF routine data/exports from staff rosters	x	x	x	x	x	x	x	x	x
Staff intent to leave (aim 2.3b)	LTCF staff	Online survey	x		x		x				

T0 to T8 refer to timepoints from baseline (T0) to start of intervention (T1, one month after T0) followed by three-monthly data collection points (T2 to T8)

*Abbreviations*: *LTCF* long-term care facility

^a^ Data is quantitative unless otherwise marked

#### Implementation outcomes (aims 1 and 2.1)

Overall fidelity to INTERCARE core components (primary outcome, aim 1) will be assessed via semi-structured interviews with LTCF leadership every three months. Two researchers (one interviewer, one note-taker) will review each minimal requirement and classify it as systematically, partially, or not (yet) implemented, yielding an overall fidelity score (percentage of minimal requirements implemented as intended) at each time point. Fidelity will be interpreted as high (80–100%), moderate (51–79%), or low (0–50%), following published cut-offs [59, 60].

For aim 2.1, fidelity will also be measured separately for each core component using: 1) online surveys of INTERCARE nurses, LTCF staff, and physicians (interprofessional collaboration and evidence-based tools); 2) structured reports from LTCF leadership (comprehensive geriatric assessment and data-driven quality improvement); 3) structured reports and observation of INTERCARE nurses’ coaching activities; 4) and Excel data sheets reporting ACP conversations with newly admitted residents, at time points defined in Table 2. Sustainability will be assessed by overall self-reported fidelity over 12 months post-implementation.

Acceptability and feasibility of selected core components will be assessed with the Acceptability of Intervention Measure (AIM) and Feasibility of Intervention Measure (FIM) [61]. INTERCARE nurses and LTCF staff will complete online surveys at T0, as well as 6 and 12 months after intervention start (T2, T4), while leadership will be surveyed at T0 and T4 (cf. Table 2).

#### Economic outcomes (aim 2.2)

Costs and cost-effectiveness will be assessed from LTCF and research group perspectives using a multi-stakeholder approach [62]. Time-driven activity-based costing (TDABC) [63] will be applied during implementation and sustainment phases. Structured REDCap [64] forms will capture time spent on each implementation activity (what was done, by whom, and for how long), separately asking for the hourly wage to calculate costs per hour for the human resources used. The structured forms will be completed by LTCF personnel (including leadership, INTERCARE nurses, and other local staff targeted by the implementation strategies) and the research group. Each LTCF will provide its service price list and information on the financial impact of hospital transfers and potentially empty LTCF beds. Cost data will be merged to estimate total intervention and implementation costs. Combined with effectiveness data on unplanned hospital transfers (aim 2.3), these will be used to calculate the incremental cost-effectiveness ratio (ICER).

#### Effectiveness and organizational outcomes (aims 2.3a, 2.3b)

The clinical outcome is the number of unplanned transfers at resident level, defined as any unscheduled transfer to the ED or hospital; planned transfers (e.g., follow-up appointments, non-emergency surgery) will be excluded [65]. LTCFs will use an Excel-based monitoring tool to track hospitalizations (admission and discharge dates, type of visit, length of stay, reason for transfer). The main indicator will be unplanned transfers per 1,000 care days, with sub-analyses separating ED visits and hospital stays; only fully anonymized data will be provided to the research team.

Organizational outcomes include staff absences (sick days relative to working days and proportion of staff with sick days) and staff turnover (monthly proportion of staff who left), assessed over time. At staff level, intention to stay and intention to leave will be measured via online surveys, and facility, socio-demographic, and professional characteristics will be collected for LTCFs, LTCF leadership, INTERCARE nurses, staff, and physicians to describe the samples.

#### Qualitative data (aim 2.1)

Qualitative data will focus on fidelity to the INTERCARE nurse core component, specifically the minimum requirement “The INTERCARE nurse coaches the teams concerning complex resident situations and acute situations,” as this is a key ingredient for successful reduction in unplanned transfers, based on the INTERCARE process evaluation [66]. An insider (emic) perspective will be captured through fidelity interviews with leadership and INTERCARE nurses. In addition, INTERCARE nurses will provide three-monthly three-day logs of their coaching activities of care staff members regarding specific clinical situations involving residents (who initiated the coaching activity, who participated, topic, form, duration, location). An outsider (etic) perspective will be obtained via go-along observations of INTERCARE nurses’ daily routines and coaching practices, conducted by a PhD student/research assistant for one working day (5–7 hours) per nurse, approximately six months after intervention start [67, 68]. Observations and informal conversations will be documented in Rapid Assessment Procedure (RAP) sheets [69].

### Data analysis

All quantitative data will be analyzed using R 4.5.x [70]. Descriptive statistics will summarize implementation, economic, and effectiveness outcomes and sample characteristics (means and standard deviations or medians and interquartile ranges for continuous variables, and frequencies and percentages for categorical variables). Multiple imputation will be considered depending on the extent of missing data [71]. Following CONSORT for cluster-RCTs [51], baseline differences between arms at the LTCF level will be presented using standardized mean differences and, where appropriate, frequencies and percentages.

For aim 1 (fidelity) and aim 2.1 (acceptability and feasibility), the non-inferiority margin is set at 15%. The non-inferiority of collaborative versus individualized support will be tested using binomial generalized linear mixed models with a random effect for LTCFs, and 95% confidence intervals; non-inferiority is confirmed if the lower confidence bound does not cross 15% below the arm II value.

A convergent mixed-methods approach will be used for aim 2.1. Qualitative data (interview notes, coaching activities reports, RAP sheets) will be analyzed using framework analysis [72, 73]. combining inductive and deductive themes to identify patterns in INTERCARE nurses’ practices and coaching approaches in acute situations. Qualitative findings will be compared with quantitative fidelity trajectories to explore fidelity moderators, and observational data will be coded in MAXQDA (https://www.maxqda.com/).

For aim 2.2, descriptive analyses will summarize intervention, implementation, and hospitalization costs by arm. A cost-effectiveness analysis will estimate the ICER, defined as the difference in costs between the two modes of delivery divided by the difference in their effects, measured as the number of unplanned hospital transfers. CEA helps identify ways to redirect resources to more cost-effective approaches [74]. We will use a multi-stakeholder perspective (i.e., LTCF and research group perspectives) and a 12-month time horizon, respecting the NICE guideline while reflecting data availability [75]. One-way sensitivity analysis and scenario analysis will be used to gauge our predictions’ level of uncertainty.

For aims 2.3a and 2.3b, the effects of the two delivery modes on unplanned transfers and staff outcomes will be analyzed using mixed-effects models analogous to those for aim 2.1, accounting for clustering at LTCF level.

### Ethical considerations

The leading ethics committee (Ethikkommission Nordwest- und Zentralschweiz) ruled that the research project does not fall under the scope of the Swiss Human Research Act (REQ-2024-00414, REQ-2024-00283), since the project was deemed an organizational quality improvement. All data will be stored confidentially on password-protected servers at the Institute of Nursing Science, University of Basel, with survey, routine, and interview data stored separately from key codes; all exports will be de-identified, and data transfer will use secure SWITCHdrive links. Each LTCF signs a contract agreeing to collect organizational data and to the participation of model implementers in surveys and interviews (Supplementary Material 4). Written informed consent will be obtained for observations and informal interviews with INTERCARE nurses (Supplementary Material 5), and consent for online surveys will be sought at survey start, highlighting voluntariness, the right to skip questions, and confidential data handling. Residents may opt out of the use of their routine administrative or assessment data, which pose minimal risk as they are collected as part of usual care (Supplementary Material 6). The research group will not be able to trace any data back to a specific resident. If our research aims are not supported, LTCF residents will continue to receive the usual standards of care. All data transfer will be handled via secure links via SWITCHdrive. The study adheres to GCP principles, the Declaration of Helsinki, ICH-GCP, or ISO EN 14155 (as applicable) and all national legal and regulatory requirements; a data steward will oversee compliance with the data management plan.

## Discussion

New care models are needed to support LTCFs in meeting current and future challenges in providing high-quality care to increasingly frail and highly care-dependent residents. Implementing such models can also help address shortages of qualified professionals by directly supporting teams, fostering competency development, enhancing motivation, and promoting fidelity to the models’ core components among staff [66]. Although the evidence base exists, few models have been successfully scaled up to other settings and contexts. The INTERSCALE study addresses this gap by further expanding the research program initiated by the INTERCARE model, specifically tailored to the Swiss context and proven effective in reducing hospitalizations. INTERSCALE applies implementation science methodology to guide scale-up, using a hybrid type III implementation design to test two delivery modes: individual vs. collaborative. This study will provide a change package of the intervention and fitting implementation strategies to drive a scale-up of this nurse-led care model in the German-speaking part of Switzerland in a feasible way, also from a cost perspective. In addition, the thorough application of implementation science methodology has the potential to build the evidence base to inform further studies aimed at scaling up complex interventions in the LTC sector.

Given the demand for organizational change needed to implement a new care model, the sample will not be representative, but rather reflect LTCFs in a position to handle such a change. With the volatile market situation for professional nurses and limited resources available, we expect turnover and changes in the setup, also after LTCFs commit to participating in the study. With the 40 LTCFs envisioned, we are slightly overpowered to address possible drop-outs. Even if we lose committed LTCFs, with the qualitative and quantitative data collected, we will be able to describe and understand in depth the degree of fidelity, as well as the barriers and facilitators. In addition, with the time-driven activity-based costing (TDABC) approach, we will be able to understand LTCFs’ activities to implement and sustain the core components. In addition to assessing the clinical effects of implementation, i.e., the number of unplanned transfers, we will gain insight into how different activities might impact sustainability.

In conclusion, INTERSCALE is part of a research program that adds to the current scientific body of knowledge by exploring scale-up of complex nurse-led interventions in the LTC sector, an understudied area in implementation science. It addresses a gap concerning the application of implementation strategies that cost-effectively support scale-up. The findings of this research will have considerable implications for guiding research groups, policymakers, and healthcare organizations on how to best support LTCFs in scaling up evidence-based interventions. Moreover, it expands the evidence on two implementation outcomes of great importance – fidelity and implementation costs – for which currently little knowledge is available, supporting the overall development of implementation science in these areas.

## Supplementary Information

Supplementary Material 1.

Supplementary Material 2.

Supplementary Material 3.

Supplementary Material 4.

Supplementary Material 5.

Supplementary Material 6.

## Data Availability

We will make key datasets and supplementary files accompanying publications openly available in appropriate digital data repositories that conform to the Fair Data principles and are maintained by a non-profit organization. In our case, we will use Zenodo as the central repository. We will share specific datasets via domain-specific public repositories whenever possible (if LTCFs agree with it).

## Abbreviations

AACTT: Action, Actor, Context, Target, Time
ERIC: Expert recommendations for implementing change
EPIS: Exploration, Preparation, Implementation, Sustainment framework
FRAME: Framework for Reporting Adaptations and Modifications
GP: General Practitioner
INTERSCALE: SCAling up an Evidence-based care model for nursing homes
INTERCARE: Improving INTERprofessional CARE for better resident outcomes
ISF: Interactive Systems Framework for Dissemination and Implementation
LTCF: Long-term care facility
LTC: Long-term care
RN: Registered Nurse
