# Supplementary material for: Sustainable improvement of interprofessional care for better resident outcomes: protocol for the INTERSCALE hybrid type III effectiveness cluster-randomized trial comparing individualized and collaborative delivery of an evidence-based care model for long-term care
Source: Implement Sci. 2026 Feb 20;21:24. doi: 10.1186/s13012-026-01489-0 (PMC13032367; doi:10.1186/s13012-026-01489-0)
Supplement: Supplementary file 1 — Supplementary Material 1. [file 13012_2026_1489_MOESM1_ESM.docx]

## Supplement 1: Minimal requirements and performance objectives for the implementation of the INTERCARE core components

Leadership team: The leadership team is defined below as the facility's senior management team and unit leaders.

Project team: The planning and implementation of the INTERCARE care model requires change processes at many levels. To enable detailed planning in which all units and decision-makers (management team) are involved, it is recommended to set up a project team that represents all persons relevant to implementation in the respective facility and meets regularly.

|  | **Core component: INTERCARE nurse** | |
| --- | --- | --- |
|  | General information:  The INTERCARE nurse assumes a clinical leadership role to improve the quality of care and review care outcomes. | - The leadership team recognizes the need to hire INTERCARE nurses and integrates them into the facility. - The leadership team supports the INTERCARE nurses in their work and in introducing their role into the facility's structures. - The project team involves the INTERCARE nurse in planning how they will implement their role in the facility. - Key persons for the facility communicate the importance of the role of the INTERCARE nurses in the team - The INTERCARE nurses have or develop relevant skills and competencies through the CAS INTERCARE to carry out their role in the facility. - The leadership team communicates the introduction of the role of the INTERCARE nurses and defines their tasks, responsibilities, and competencies in the facility. - The INTERCARE nurses introduce themselves to internal and external colleagues and specialists. - The INTERCARE nurses receive feedback regarding their performance in their role in the facility. The project team decides who should give the feedback (e.g., physicians, nurse experts, peer-to-peer feedback among INTERCARE nurses). |

|  | **Core component: INTERCARE nurse** | |
| --- | --- | --- |
| No. | **Minimal requirements (adapted for INTERSCALE)** | **Performance Objective (recommendation)** |
| 1 | The INTERCARE nurse holds at least a nursing diploma and has worked in clinical geriatrics for at least 3 years. | - The INTERCARE nurse ("internal" or "external" applicant) goes through a recruitment process. - The INTERCARE nurse submits certificates (reference to professional experience). - The INTERCARE nurse signs an employment contract with the facility. |
| 2 | The INTERCARE nurse is present in the units responsible for at least 4 days a week and is available to support the teams. | - The leadership team ensures that the tasks of the INTERCARE nurse are feasible in the time available and provides sufficient time resources for their tasks. - The INTERCARE nurse, together with their supervisors and the unit leaders, develops a work plan that outlines designated time slots for regular exchanges with each unit. - The INTERCARE nurse introduces themselves and their role to the units they are responsible for. - The INTERCARE nurse works on-site at the facility according to an agreed work schedule with a minimum of four days (full day not mandatory) per week in the units they are responsible for |
| 3 | The INTERCARE nurse coaches the teams concerning complex resident situations and acute situations. | - The INTERCARE nurse communicates their availability for coaching upon the introduction of their role. - The INTERCARE nurse identifies the need for coaching during their daily rounds on the units, by observing the care team and in discussions with them - The INTERCARE nurse encourages the care teams to ask for coaching when needed actively - The INTERCARE nurse uses coaching techniques when carrying out bedside coaching (i.e., supporting staff in direct care of residents). - The INTERCARE nurse evaluates the coaching situations with the care team involved. |

|  | **Core component: INTERCARE nurse** | |
| --- | --- | --- |
| No. | **Minimal requirements (adapted for INTERSCALE)** | **Performance Objective (recommendation)** |
| 4 | The INTERCARE nurse assesses residents in acute and complex situations according to their skills and knowledge | - The INTERCARE nurse defines acute situations in which they should be involved and informs the care team accordingly - The care team calls the INTERCARE nurses in acute situations. - The INTERCARE nurse goes directly to the corresponding unit after the call. - The INTERCARE nurse contacts the care team of the unit to discuss the situation. - The INTERCARE nurse assesses residents, using specific assessment tools if necessary, and interprets the assessment. - The INTERCARE nurse discusses the situation with the care team and provides support in clinical decision-making. - The INTERCARE nurse supports the care team in organizing and prioritizing the necessary tasks in acute resident situations. If necessary, the INTERCARE nurse performs the required tasks themselves (e.g., contacting the physician). - In complex resident situations, the INTERCARE nurse obtains a comprehensive picture of the resident, involving the direct care team and, if necessary, a broader interprofessional team. - The INTERCARE nurse leads or supports clinical decision-making and the development and implementation of an (interprofessional) treatment plan. |
| 5 | The INTERCARE nurse discusses and reflects on resident situations together with the care team within a defined framework to increase their clinical skills (e.g., case discussions, hospital reflections). | - The INTERCARE nurse identifies needs for support concerning the clinical skills of the care team. - The INTERCARE nurse discusses with the management team the identified needs for support concerning the clinical skills of the care team. - The INTERCARE nurse defines the context in which the care team wishes to increase clinical competencies (e.g., case discussions, hospital reflections). - The INTERCARE nurse decides how the format of the meetings should look: when, where, and with whom. - The INTERCARE nurse conducts the meetings and reflections with the care team. - The INTERCARE nurse evaluates and documents the meetings and reflections in accordance with a previous internal agreement. |

|  | **Core component: Interprofessional collaboration** | |
| --- | --- | --- |
|  | General information:  The interprofessional team is defined as at least two different professional groups, such as nursing staff, physicians, pharmacists, pastoral care workers, representatives of therapeutic professions, or social workers, who work together to address the needs of the residents. | - The professionals involved recognize the value of interprofessional collaboration. - The project team, in collaboration with the INTERCARE nurse, defines the tasks needed for the implementation of this core component. |
| No. | **Minimal requirements  (adapted for INTERSCALE)** | **Performance Objective (recommendation)** |
| 1 | The project team establishes a structure that promotes interprofessional communication (e.g., regular meetings) between at least two different professional groups. | - The project team plans which professionals are responsible for ensuring interprofessional communication. - The project team decides which exchange formats should be used to improve interprofessional communication. - The project team determines which professionals should be involved in interprofessional communication. - The project team, in collaboration with the management team, reviews the current communication structures of the facility and adapts them to facilitate interprofessional communication. Guidelines for preparing and conducting meetings or rounds are also revised if necessary. - The project team determines the extent to which the INTERCARE nurse is involved in the visits with the physicians (e.g., regularly or upon request). - The project team timely informs all professionals involved regarding tools and structures that promote interprofessional communication. - The project team informs or reminds all professionals involved of the benefits of interprofessional communication. - The project team promotes communication by designing user-friendly structures (e.g., by creating e-mail templates, organizing regular meetings, etc.). |
| 2 | All qualified nursing staff (i.e., registered nurses, licensed practical nurses) who are in contact with residents and notice a change in their state of health should contact the responsible healthcare professional. | - The project team clarifies which responsible healthcare professionals are available at the facility (e.g., physicians, podiatrists, wound experts). - The project team determines or makes suggestions about how and in which situations contact should be made (e.g., STOP&WATCH, telephone, email, or via the INTERCARE nurse) - The project team defines, documents, and communicates the exact communication process |
| 3 | If there are changes in the residents’ health conditions, the assessment results are interpreted and, if necessary, the resident's treatment plan is adapted by an interprofessional team (at least two healthcare professionals, one of whom is a nurse). | - The changes in the residents’ health conditions are discussed by the responsible care team (at least one registered nurse) within the interprofessional team and, where possible, with the resident themselves and/or their relatives. - Possible changes to the treatment plan and therapy goals are made based on the findings from the interprofessional discussion (e.g., Comprehensive Geriatric Assessment (CGA)) and agreed by all those involved (including the resident and relatives). |
| 4 | The INTERCARE nurse supports the communication process between the physician and the nursing staff. | - The project team, together with the INTERCARE nurse, determines the level of involvement of the INTERCARE nurse (i.e., whether they are involved in visits or not) - The INTERCARE nurse has an overview of both internal and external interprofessional communication structures in the facility. - The INTERCARE nurse oversees and ensures that all individuals involved are familiar with the communication structures set up by the project team (e.g., ISBAR) and use them appropriately. - All individuals involved are informed that the INTERCARE nurse can be consulted in case communication problems occur and that they will involve the management team if needed. - The INTERCARE nurse, if necessary, together with representatives of the management team, leads critical conversations in the interprofessional team |

|  | **Core component: Advance Care Planning (ACP)** | |
| --- | --- | --- |
|  | General information:  The existence of a living will, and the discussion of residents' wishes regarding resuscitation, possible hospitalization, and antibiotic therapy are clarified for each newly admitted resident (medical emergency order), and, if necessary, a treatment plan is developed | - The project team ensures that the specialists involved have the necessary knowledge about ACP. |
|  | Core component: Advance Care Planning (ACP) | |
| No. | **Minimal requirements  (adapted for INTERSCALE)** | **Performance Objective (recommendation)** |
| 1 | The project team creates structures, processes, and tools to implement ACP, by also integrating input from the physicians the facilities work with. | - The leadership team informs all professionals involved about the benefits of ACP for the facility and the planned implementation. - The project team clarifies internally which employees are responsible for implementing ACP, which tasks are to be fulfilled, and how the INTERCARE nurse is involved. - The project team clarifies in advance the manner of cooperation with the physicians (GPs and on-site physicians) and the time resources needed, as well as ensures that these processes are adhered to - The project team promotes access to a palliative care network (external specialists or institution). |
| 2 | For each newly admitted resident, the following aspects must be recorded in the resident documentation by the responsible person(s):   - Decision regarding resuscitation - Decision regarding hospitalization - Decision regarding use of antibiotics for infections | - The project team clarifies the need for and organizes training courses for the employees involved to strengthen their ability to conduct confident ACP conversations in difficult situations - At least one employee attends the ACP introductory course. - The leadership team provides sufficient time resources for the responsible person to conduct discussions with residents. - Physicians are involved (if necessary) in the initial consultation and in the follow-up discussions with residents and relatives. - The project team ensures that the residents and their relatives are involved in the decision-making process. |
| 3 | For residents in an unstable condition before weekends/holidays, the responsible person(s) clarify treatment plans and medical emergency orders. | - The project team defines, if possible, together with the physician, which resident situations are to be considered acute or unstable. - The project team, together with the responsible person(s), defines structured steps and develops decision-making aids for emergency situations, which are practiced using case studies/demonstrations. - The project team ensures that all care teams are informed about the guidelines for emergency situations. |

|  | **Core component: Evidence-based tools (EBT) - STOP&WATCH** | |
| --- | --- | --- |
|  | General information:  The introduction of the **STOP&WATCH** tool promotes the flow of information between nursing assistant staff (nurse aides, healthcare assistants) and daily supervisors at the unit, regarding changes in the condition of residents | - The leadership team ensures there is a buy-in in the project team regarding why STOP&WATCH is beneficial for the facility. - The professionals involved in the implementation of STOP & WATCH recognize its value in strengthening the role of all formal caregivers. |
|  | Core component: Evidence-based tools (EBT) - STOP&WATCH | |
| No. | **Minimal requirements  (adapted for INTERSCALE)** | **Performance Objective (recommendation)** |
| 1 | The project team creates a plan and appropriate structures for the integration of STOP&WATCH, and clarifies the INTERCARE nurse's responsibility for implementing the tool. | - The leadership team ensures that sufficient resources are available to introduce STOP&WATCH in the unit. - The project team informs all employees that STOP&WATCH is to be introduced in the unit. - The project team develops a work plan tailored to the facility's resources:   - Determines which employees use STOP&WATCH: Nursing assistants are expected to use it, but other employees could use it, too   - Specifies the person(s) from whom the documentation of STOP&WATCH is collected.   - Provides instructions for documenting the STOP&WATCH results.   - Provides guidance on the next steps or actions (appropriate assessment) to be taken following the STOP&WATCH results - The leadership team ensures that sufficient staff are available to implement STOP&WATCH as planned. - The INTERCARE nurse trains the responsible staff in the application and documentation of STOP&WATCH in daily practice. - The INTERCARE nurse distributes the pocket version of the STOP&WATCH (e.g., during training). |
| 2 | The INTERCARE nurse ensures the integration of STOP&WATCH into daily practice. | - The INTERCARE nurse ensures the introduction of STOP&WATCH into daily practice. - The INTERCARE nurse instructs the nursing assistant staff in the use of the STOP&WATCH tool. - The INTERCARE nurse ensures that those involved know where and from whom the documents are collected. - The INTERCARE nurse implements STOP&WATCH in every participating unit. - Employees in specific roles, such as champions or unit leaders in the units, support the INTERCARE nurses in the introduction of STOP&WATCH. - The INTERCARE nurse reviews the application of STOP&WATCH and provides feedback to those involved. |

|  | **Core component: Evidence-based tools (EBT) - ISBAR** | |
| --- | --- | --- |
|  | General information:  The introduction of the **ISBAR** tool promotes the structuring and improvement of communication between the healthcare professionals/qualified nursing staff and the physicians, regarding changes in the condition of residents | - The leadership team ensures there is a buy-in in the project team regarding why ISBAR is beneficial for the facility. - The professionals involved in the implementation of ISBAR recognize its value in strengthening the role of all formal caregivers. |
|  | Core component: Evidence-based tools (EBT) - ISBAR | |
| No. | **Minimal requirements  (adapted for INTERSCALE)** | **Performance Objective (recommendation)** |
| 1 | The project team creates a plan and appropriate structures for integrating ISBAR, and clarifies the INTERCARE nurse's responsibility for implementing the tool. | - The project team develops a work plan tailored to the facility's resources: - The leadership team informs all employees that ISBAR is used in all communication with physicians or the INTERCARE nurse. - The project team determines the practical assessments for the use of ISBAR (e.g., pocket version, e-mail structure, telephone structure). - The leadership team ensures that sufficient staff members are available to implement ISBAR as planned. - The INTERCARE nurse trains the nursing staff in the use of ISBAR. - The INTERCARE nurse provides the nursing staff with the practical tools needed to use ISBAR. |
| 2 | The INTERCARE nurse ensures the integration of ISBAR into daily practice. | - The INTERCARE nurse plans the necessary steps to introduce ISBAR in each participating unit within the first 6 months and ensures that these steps are implemented. - Employees in specific roles, such as champions or unit leaders, support the INTERCARE nurse in introducing ISBAR. - The INTERCARE nurse plans and clarifies how to document the use of ISBAR in each participating unit. |

|  | **Core component: Comprehensive Geriatric Assessment (CGA)** | |
| --- | --- | --- |
|  | General information:  An interdisciplinary process is introduced to assess residents’ physical condition, mental health, functional abilities, and socio-economic circumstances, as well as their values and resources. | - Everyone involved recognizes the value of the CGA for their daily work. |
|  | Core component: Comprehensive Geriatric Assessment (CGA) | |
| No. | **Minimal requirements (adapted for INTERSCALE)** | **Performance Objective (recommendation)** |
| 1 | A CGA working group is put together to implement this core component. | - The project team determines who the members of the CGA working group are, who are responsible for implementing this core component, and where the INTERCARE nurse has an integral role. - The CGA working group meets regularly to discuss the implementation of the CGA. |
| 2 | The CGA working group defines a CGA concept adapted to the facility, based on the research team's model. | - It is recommended that the CGA concept be drawn up within the first six months of implementation. - The CGA working group takes the lead in developing the CGA concept. - The CGA working group evaluates which assessments (regarding all five geriatric dimensions: physical, mental, functional, socio-economic, values and resources) are available in the facility, are used by which professional group, and in which context. This also considers which instruments the INTERCARE nurse should use. - The CGA working group also considers unplanned transfers and key clinical domains / geriatric syndromes (e.g., mobility, cognition, skin, incontinence) that are frequently addressed. - The CGA working group is familiar with possible CGA assessments provided by the research group. - The CGA working group critically assesses and chooses a reasonable number of assessments to implement, depending on the goals of the facility - The CGA working group selects the assessments for each geriatric dimension to be used in the facility (e.g., dementia, palliative care). - The following points are defined for each selected assessment: - What qualifications should the users have? - To what extent is coaching required for the assessments? - Who uses the assessment? - What triggers its use, when, and in what situations is it used? - How is the assessment used and evaluated? - How are the results documented? - Who interprets the results? - Who follows up on the results? - What are the triggers for a reassessment? - The CGA concept includes a role description for the INTERCARE nurses in the CGA process. - The CGA working group guides what the following steps are to be taken, depending on the assessment results. The INTERCARE nurse ensures residents and relatives are involved in the decision-making process and in the preparation of the treatment plan. |
| 3 | The facility implements the CGA concept in accordance with an individually defined plan. | - The CGA working group draws up a project plan for the introduction of the CGA concept, based on what resources are available and what timetable is feasible - The CGA working group conveys the importance and benefits of the selected assessments and addresses the different levels of employees. - The CGA working group informs and instructs employees on the following: - The situations in which an assessment should be carried out. - The way the assessment should be conducted - The documentation of conducted assessments and their results. - The interpretation of assessment results, in particular the clinical decision-making and the estimation of possible trajectories that may lead to acute situations, is carried out as needed within the interdisciplinary team. - The procedure for how the employees conducting the assessments receive feedback. - The CGA working group / designated person plans for and evaluates the implementation of the CGA concept. |
| 4 | The CGA working group evaluates the collected data (see below, DDQI) twice a year to determine additional possible uses of CGA assessments to prevent unplanned transfers. | - The CGA working group identifies topics from the reflections of the transfer data that can be addressed via CGA assessments. - The CGA working group informs the care teams about any modifications to the CGA concept that may arise from the reflection on transfer data. |

|  | **Core component: Data-driven quality improvement (DDQI)** | |
| --- | --- | --- |
|  | General information:  This core component includes the systematic collection and analysis of data on hospital or emergency department transfers, as well as other quality-improvement data. | - The leadership team recognizes the value of data-driven quality improvement. - The professionals involved recognize the need for a joint effort to achieve the quality objectives of the facility. |
|  | Core component: Data-driven quality improvement (DDQI) | |
| No. | **Minimal requirements  (adapted for INTERSCALE)** | **Performance Objective (recommendation)** |
| 1 | The INTERCARE nurse completes a reflection tool after every unplanned hospital transfer and initiates team reflections. | - The project team informs the unit leader and the care team about the introduction of the reflection tool. - The project team defines a process for dealing with the reflection tool, including: - The INTERCARE nurse completes a reflection tool after every unplanned transfer. - After every unplanned transfer, they conduct a guided team reflection with the care team involved in the transfer. - The INTERCARE nurse integrates the data collected from the reflection process with the team into the reflection tool. |
| 2 | The facility continuously collects data on all hospitalizations and emergency department visits, as well as other quality improvement data. | - The leadership team determines who is responsible for collecting the data. - The leadership team determines which data/topics they would like to monitor for data-based quality improvement. This includes at least hospitalizations (at least 1 overnight stay in hospital) and emergency department admissions. - The project team determines whether the quality improvement should take place at the unit or facility level or whether individualized quality development is necessary at the unit level (e.g., internal topic of pain). - The project team determines who collects the data and how often the data should be collected. - The project team prepares the nursing staff to be involved in data-based quality improvement efforts via a format that enables exchange. They highlight the benefits of data-based quality improvement, discuss concrete implementation, and address questions. - The leadership team gives the responsible persons access to existing software programs for data collection. - The designated person collects the data and prepares it for evaluation. |
| 3 | The facility interprets the collected data, determines which goals/topics they want to work on to improve quality, and identifies the measures required to achieve those goals. | - The project team determines which persons are involved in the interpretation and discussion of the data. - The leadership team ensures that the persons responsible for data collection and interpretation are prepared for these tasks. - The person responsible plans meetings to discuss and interpret the collected data. - The person responsible distributes an overview of the collected data to the persons involved in the evaluation. - During the planned meetings, the people involved interpret the data and determine the topics/goals they want to work on to improve quality. - During the meetings, the people involved determine the measures required to achieve the objectives and discuss whether a PDCA cycle would be helpful. |
| 4 | The facility conducts a PDCA cycle for at least one of the selected objectives/topics (see the *previous requirement).* | - The project team determines in advance a person or group (e.g., the responsible persons from point 3 above) who is/are responsible for the implementation of the PDCA cycle. - The level at which the PDCA cycle is to be carried out (facility or unit level) is determined in advance. - The PDCA cycle is implemented. - Positive results of conducting a PDCA cycle are emphasized within the facility. |
