# Supplementary material for: Sustainable improvement of interprofessional care for better resident outcomes: protocol for the INTERSCALE hybrid type III effectiveness cluster-randomized trial comparing individualized and collaborative delivery of an evidence-based care model for long-term care
Source: Implement Sci. 2026 Feb 20;21:24. doi: 10.1186/s13012-026-01489-0 (PMC13032367; doi:10.1186/s13012-026-01489-0)
Supplement: Supplementary file 4 — Supplementary Material 4. [file 13012_2026_1489_MOESM4_ESM.zip › Supplement 4_Appendix_A_data collection_260214_ESM.docx]

# Appendix A: Data collection

Version 1.0 / Month Year / x. cohort Month/Year [baseline month] – Month/Year [end sustainment phase]

The total study period is from (T0) to (T8)

|  | **Type of data collection** | **Time required per data collection point and over the entire duration of the study** | **Source of data collection** | **Topics/content of the collection** | **Declaration of consent required** | **Data collection points over the entire duration of the study^a^** | | | | | | | | |
| --- | --- | --- | --- | --- | --- | --- | --- | --- | --- | --- | --- | --- | --- | --- |
|  |  |  |  |  |  | **T0** | **T1** | **T2** | **T3** | **T4** | **T5** | **T6** | **T7** | **T8** |
| **1)** | **LTCF** | | | | | | | | | | | | | |
| **1)** | Structured data export of routinely collected/administrative **organizational data on personnel**^b^ | Approx. 2h per data collection point  A total of 16 hours per LTCF | Internal personnel planning system, format is agreed with the LTCF | - Employee absences due to illness - turnover | Consent is in the contract between the [UNIVERSITY] and the LTCF | Three-monthly reporting from T1 | | | | | | | | |
| **2)** | **LTCF management** | | | | | | | | | | | | | |
| **2a)** | Structured form for reporting on the **implementation of** **two core elements** | Approx. 1h per data collection point  Total 1h per LTCF | Short questionnaire in a Word document | - Degree of implementation fidelity for two core elements: Multidimensional Geriatric Assessment (CGA) and Data-Driven Quality Improvement (DDQI) | Consent is regulated in the contract between the [UNIVERSITY] and the LTCF |  |  |  |  | x |  |  |  |  |
| **2b)** | Questionnaire/online form for reporting on the **activities of the LTCFs in connection with the implementation strategies** (to be able to calculate the costs of implementation) | Approx. 2h per month (effort decreases with time)  A total of 48 hours per LTCF (this includes all persons who implement implementation strategies) | REDCap® questionnaire (online) | - Recording the implementation of the implementation strategies provided by the study team, including time spent (e.g., coaching, workshops) - Recording of implementation strategies developed and implemented by the LTCFs in addition to the strategies provided by the study team (if applicable, e.g., training, information events) | Consent is regulated in the contract between the [UNIVERSITY] and the LTCF | Monthly reporting to the study team | | | | | | | | |
| **2c_1)** | Questionnaire on **the characteristics of the LTCF** | Approx. 40 min. per data collection point  Total 40 min. per LTCF | REDCap® questionnaire (online) | - Size of the facility, number of units, staffing of the units, focus, medical model, etc. (T0) | Consent is regulated in the contract between the [UNIVERSITY] and the LTCF | x |  |  |  |  |  |  |  |  |
| **2c_2)** | Questionnaire on the **characteristics of leadership** | Approx. 20 min. per data collection point  Total approx. 40 minutes per LTCF, depending on the number of people completing the survey | REDCap® questionnaire (online) | - Acceptance and feasibility of the INTERCARE model (T0 & T4) - Upper management: Professional background, duration of employment in the LTCF (repeated in T4 only if there is a change in personnel) | A written declaration of consent will be obtained from all participants prior to the survey | x |  |  |  | x |  |  |  |  |
| **2d)** | Questionnaire on **prices, costs, and revenues of the companies** | Approx. 1h per data collection point  Total 2h per LTCF | Word document or pdf uploaded via SWITCHdrive | - Price list/tax regulations for residents - Information on the impact of hospital admissions of residents on the costs/income of the LTCF - Wage of the INTERCARE nurse and wage of the other persons involved in the study | Consent is regulated in the contract between the [UNIVERSITY] and the LTCF |  | x |  |  |  | x |  |  | x |
| **2e)** | Focus groups for **process evaluation of implementation strategies**^d^ | Approx. 2h per data collection point  A total of 4 hours per participant (LTCF management, INTERCARE nurse, project managers, etc.: max. 2 persons per LTCF) |  | - Perception of how implementation strategies bring about change - Awareness of local contextual factors that influence implementation strategies | A written declaration of consent will be obtained from all participants prior to the focus group |  |  |  |  | x |  |  | x |  |
| **2f)** | Semi-structured interviews to measure **fidelity to the core elements of the INTERCARE model**^c^ | Approx. 1h per data collection point  A total of 9 hours per participant (LTCF management and/or INTERCARE nurse) |  | - Status of implementation of the core elements (intervention fidelity, hindering & facilitating factors) | Consent is regulated in the contract between the [UNIVERSITY] and the LTCF | x | x | x | x | x | x | x | x | x |
| **2g)** | Semi-structured interviews on **experiences with the implementation strategies** (a result of the process evaluation)^d^ | approx. 1.5h per data collection point  A total of 3 hours per participant (depending on the LTCF: LTCF management/project manager / INTERCARE nurse) |  | - Experience with the implementation strategies developed by the study team | A written declaration of consent will be obtained from all participants prior to the interview |  |  |  |  | x |  |  |  | x |
| **3)** | **INTERCARE nurse** | | | | | | | | | | | | | |
| **3a)** | Structured form for reporting **on coaching activities** | approx. 1.5h per data collection point  Total 12h per participant | Word document (structured) | - Implementation of coaching activities for complex resident situations and situations with relatives with the care staff | Consent is regulated in the contract between the [UNIVERSITY] and the LTCF |  | x | x | x | x | x | x | x | x |
| **3b)** | Questionnaire on **professional background, quality of working environment, personnel outcomes, perceived quality of care, fidelity, as well as acceptance and perceived feasibility of the model** | approx. 1h per data collection point  Total 3h per participant | REDCap® questionnaire (online) | - Professional background, length of employment in the LTCF, information on experience as an INTERCARE nurse (T0 only) - Quality of working environment, personnel results (e.g., job satisfaction, intention to quit) - Perceived quality of care - Acceptance and feasibility of the INTERCARE model - Fidelity to the core element of interprofessional collaboration | Written consent is obtained before the questionnaire is completed | x |  | x |  | x |  |  |  |  |
| **3c)** | Observation and informal survey to measure **fidelity in relation to coaching and empowerment activities** | 1 day, including approx. 1h informal discussion  Total 6h per participant |  | - Recording how the INTERCARE nurse performs various tasks (i.e., implementation of minimum requirements), organizes and structures coaching activities, and deals with acute situations, etc. | A written declaration of consent will be obtained from all participants prior to the observation |  |  | x |  |  |  |  |  |  |
| **4)** | **Other staff in direct care and support** | | | | | | | | | | | | | |
| **4)** | Questionnaire on **professional background, quality of working environment, personnel outcomes, quality of care, acceptance and feasibility of the INTERCARE model, fidelity to core elements of INTERCARE** | approx. 20 min. per data collection point  Total 1h per participant | REDCap® questionnaire (online) | - Professional background - Quality of working environment, personnel outcomes (e.g., job satisfaction, intention to quit), - Perceived quality of care - Acceptance and feasibility of the INTERCARE model - Fidelity to evidence-based instruments and interprofessional collaboration | Written consent is obtained prior to the survey | x |  | x |  | x |  |  |  |  |
| **5)** | **Physicians** | | | | | | | | | | | | | |
| **5)** | Questionnaire on **professional background and fidelity to the core elements** | approx. 5-10 min. per data collection point  Total 10-20 min. per participant | REDCap® questionnaire (online) | - Professional background, number of residents per establishment for whom the physicians are responsible - Fidelity to the core elements (including fidelity to evidence-based instruments and interprofessional collaboration) | Written consent is obtained before the questionnaire is completed |  |  | x |  | x |  |  |  |  |
| **6)** | **Residents** | | | | | | | | | | | | | |
| **6a)** | Structured data export of **routinely collected/administrative resident data** | Approx. 15 min. per data collection point  Total 2 hours per LTCF | Only in LTCFs with RAI-NH  In LTCFs with BESA, the export is with BESA QSys  Alternative: Export from the BESA QSys dashboard | - National quality indicators (pain, malnutrition, polypharmacy, physical restraint use) incl. variables for risk adjustment: care level, interRAI Cognitive Performance Scale (CPS), life expectancy, interRAI Depression Rating Scale (DRS) | Completely anonymized data is recorded for all residents.  This data is used only for benchmarking among participating LTCFs, not for study purposes. | Three-monthly reporting from T0 | | | | | | | | |
| **6b)** | Structured data export on **visits to emergency departments and hospital admissions of residents**^b^ | Approx. 3h at the beginning of the study to define and prepare the format for data collection  approx. 30 min./ per month for compiling the data | Excel data form or internal systems, format is agreed with LTCFs | - Forwarding of de-identified data for all residents who visited the emergency department or were admitted to the hospital (e.g., date of transfer, planned or unplanned, length of stay, reason for hospitalization, type of hospital, reflection tool completed) | All residents who do not wish their data to be shared may decline to have their details included in the study (opt-out). | Three-monthly reporting from T1 | | | | | | | | |
| **6c)** | Structured form for reporting on the fidelity **of implementation of Advance Care Planning (ACP)**^b^ | approx. 5 min./per resident | Excel data form | - Implementation of the core element of ACP for new residents: Existence of the elements of a medical emergency order; i.e., consultation with residents and/or relatives regarding life-prolonging measures/symptom relief (e.g., in relation to resuscitation, hospitalization, administration of antibiotics, and/or intensive medical treatment) | All residents who do not wish their data to be passed on have the opportunity to opt out of having their details included in the study (opt-out option). | One-off reporting for each new resident as soon as the first medical emergency order or treatment plan with basic questions for residents has been completed | | | | | | | | |
