# Supplementary material for: Sustainable improvement of interprofessional care for better resident outcomes: protocol for the INTERSCALE hybrid type III effectiveness cluster-randomized trial comparing individualized and collaborative delivery of an evidence-based care model for long-term care
Source: Implement Sci. 2026 Feb 20;21:24. doi: 10.1186/s13012-026-01489-0 (PMC13032367; doi:10.1186/s13012-026-01489-0)
Supplement: Supplementary file 4 — Supplementary Material 4. [file 13012_2026_1489_MOESM4_ESM.zip › Supplement 4_0 Contract INTERSCALE_251228_ESM.pdf]

INTERSCALE cooperation agreement

from Month, Day, Year

between the [Research team], represented by [PI], hereinafter referred to as "**[university]**".

and

[company], represented by [LTCF leader], hereinafter referred to as "the **LTCF"**.

Preamble

This agreement regulates the rights and obligations of the contracting parties within the framework of cooperation in INTERSCALE.

Object and scope of the order

The subject of the contract is the INTERSCALE Study, Work Package 2 (Implementation of INTERCARE), hereinafter referred to as the "**Study**". The data collection to be provided by the parties, details of the data collection, and a schedule for the implementation of the Study are set out in Annex A (Data Collection)
B (study planning) and C (project schedule).

Implementation of the study

The LTCF agrees to carry out the study in accordance with the present compilation of the data collection, version 1.0 dated [Month, Year] (Annex A), the associated study planning, version 1.0 dated [Month, Year] (Annex B), and the project schedule, version 1.0 dated [Month, Year] (Annex C). The three annexes are an integral part of this contract. The compilation of the data collection also regulates, in particular, which data will be collected within the scope of this contract and for which data an additional declaration of consent from the participants is required. The study's implementation will be monitored and coordinated on-site by [project management].

INTERSCALE is a cluster-randomized controlled trial. This means that the study team randomly assigns the participating LTCFs to one of two arms, each with a specific set of implementation strategies. The LTCF agrees to participate in the study in the randomly assigned arm. It is not possible to change the assigned arm of the study.

The parties shall provide each other with the information required to conduct the study and make the necessary documents available in good time.

Remuneration

The participating LTCFs receive compensation equal to two-thirds of the participation costs for the INTERCARE Certificate of Advanced Studies (CAS) course offered by the [university] for one nursing specialist per LTCF, provided the nursing specialist attends the CAS during the implementation of the study. No compensation will be paid if a nurse from the LTCF starts the CAS after the end of the intervention phase in the LTCF.

Term and right of termination

This contract comes into force upon the complete and legally valid signature of both parties, as of the date on the first page of this contract, and ends upon completion of the study. The planned completion of the study is noted in Appendix B, Study Planning. The provisions of the sections on the study results and data confidentiality, as well as on publications, shall continue to apply after termination of the contract.

The contract may be terminated prematurely by either party before the end of the contract period, provided that written notification is given 60 days before the end of the cooperation.

Study results and confidentiality of data

Data routinely collected by the facility using needs assessment instruments (RAI-NH/BESA) or care documentation systems is the property of the LTCF and is made available to the [university] as part of the study. All other data collected during the study period is the property of the [university] if no opt-out declaration from residents or their legal representatives is available.

The parties agree that the use and disclosure of health-related data and medical information is subject to Swiss data protection regulations. Both parties, therefore, make sure to take all necessary measures to protect the identity of the residents' health data

and medical information and to comply with the relevant legal provisions in the area of data protection

The INTERSCALE study was submitted to the Ethics Committee of Northwestern and Central Switzerland (EKNZ). The EKNZ declared for all cantons of German-speaking Switzerland that the study does not fall within the scope of the Human Research Act (Req-2024-00283: the collection of data at the organizational level and on implementation results is not research on diseases or the structure of the body, according to HFG Art. 2 para. 1/ Req-2024-00414: The collection of data from residents constitutes a quality assurance project that does not require ethics committee approval).

Publications

The [university] has the lead in all scientific publications on the study. The LTCF may participate in scientific publications if it so wishes. For the determination of possible authorship by employees of the LTCF in a scientific journal, we adhere to JAMA's operationalization of the COPE (Committee on Publication Ethics) guidelines.

Results should only be published by the LTCF, for example, in the local press, in consultation with the [university], or based on written [university] reports on the study.

General regulations

This contract governs the parties' relationship with respect to the study alone. There are no ancillary agreements in this regard. Any previous agreements are hereby terminated. Amendments and additions to this contract must be made in writing and signed by all parties.

Should any provision of this contract be invalid or become impossible to fulfill, this shall not affect the validity of the remaining provisions. In this case, the LTCF and [university] undertake to immediately replace the invalid provision with a permissible, valid agreement that comes closest to the original intention in terms of its content. The same applies in the event of a contractual gap.

The parties agree that general information regarding the type of cooperation (area of collaboration, identity of the contracting parties) may be made public.

Under this contractual relationship, the parties do not acquire any rights from the other party beyond those expressly granted in this contract.

Rights and obligations arising from this contract may not be transferred to third parties without the written permission of the other party. Such permission shall not be unreasonably withheld.

This contract is subject to Swiss law. The place of jurisdiction shall be the ordinary courts of Basel.

Signatures

LTCF

I have read this contract, including the appendices, and accept the obligations it imposes.

XXX	Place and date

[University]

I have read this contract and accept the obligations arising from it

[PI]	Place and date

Attachments:

Appendix A: Data collection

Appendix B: Study planning

Appendix C: Project schedule
