# Supplementary material for: Sustainable improvement of interprofessional care for better resident outcomes: protocol for the INTERSCALE hybrid type III effectiveness cluster-randomized trial comparing individualized and collaborative delivery of an evidence-based care model for long-term care
Source: Implement Sci. 2026 Feb 20;21:24. doi: 10.1186/s13012-026-01489-0 (PMC13032367; doi:10.1186/s13012-026-01489-0)
Supplement: Supplementary file 4 — Supplementary Material 4. [file 13012_2026_1489_MOESM4_ESM.zip › Supplement 4_Appendix_B_study planning_260214_ESM.docx]

# Appendix B: Study planning

Version 1.0 / Month Year / x. cohort Month/Year [start preparatory phase] – Month/Year [end sustainment phase]

| PROJECT TASK | DESCRIPTION | RESPONSIBLE PERSONS | PERSONS INVOLVED | TIME PERIOD | Responsibility | |
| --- | --- | --- | --- | --- | --- | --- |
|  |  |  |  |  | INTERSCALE study team |  |
| Preparatory phase [2 months] | | | | | | |
| Employment INTERCARE nurse | a) Contractual employment of the INTERCARE nurse and clarification of the exact scope of tasks and responsibilities (also concerning sufficient job percentages)  b) Clarify participation in INTERCARE CAS  c) The INTERCARE nurse introduces itself to internal and external colleagues in its new role | Management team | One or more INTERCARE nurses | Until the start of the intervention phase | Provision of job description and job advertisement | Going through the application process; defining tasks and responsibilities together |
| Define the INTERSCALE project structure and the project team | Clarify internally how the project structure is organized and which persons are to be integrated into the project team to plan the project implementation | team | Depending on the LTCF | Compilation in [preparatory month 1], possibly expanded after the preparatory workshops | Recommendations on which persons in the LTCFs can be involved | Setting up the INTERSCALE project structure and the INTERSCALE project team |
| Preparatory workshops | Introduction to the model, tasks, responsibilities, and procedures during the study | Management team, members of the project team, INTERCARE nurse | (if possible) entire project team | [preparatory month 1]: 1 workshop in presence (half day);  3 online workshops in [preparatory months 1 and 2] (half day each) | Preparation, organization, implementation | Participate  Follow-up according to the tasks and documents provided in the workshops,  Implement what you have learned |
| Analysis of the implementation conditions (context analysis) | Discuss factors that hinder and promote the implementation of the individual core elements | Management team  INTERCARE nurse  Unit leaders  Project team | Persons responsible from the previous column in discussions with various staff groups, depending on the LTCF, e.g., therapy staff, physicians, persons responsible for needs assessment tools, nurse experts, quality managers, etc. | [preparatory months 1 and 2] | Preparation and guidance of the context analysis within the preparatory workshops | Carry out an analysis of the context |
| Creation of an LTCF-specific project plan | Create project plan | Project team | Project team, possibly administration, others | Until the end of [preparatory month 2] | Provision of study planning and project schedule for estimating resources and necessary time windows. Provision of a template for project planning. | Create detailed project planning |
| Adaptation of the model to the LTCF | The INTERSCALE project team prepares an introduction using the implementation handbook (adaptation of the model to the LTCF, adaptation of internal processes, e.g., in connection with data collection).  Minimum requirements of the core elements must be introduced; peripheral elements and action plans should be considered in the implementation, depending on the context | Project team | Entire project team, management team, INTERCARE nurse, administration, and possibly other persons | Preparatory workshops accompany the process | Preparation,  Assistance,  Develop documents (guidelines, tools, information...) | Adaptation of structures and processes according to workshops |
| Prepare introduction of the reflection tool | Reflection on hospital admission | Project team, unit leaders, INTERCARE nurse | Care teams, depending on the structure | Start at the beginning of the baseline survey with reflections on hospital admission | Provide a tool for reflection | Train staff;  Define guidelines; implement |
| Inform all employees of the LTCF about the INTERSCALE project and the introduction of the INTERCARE model | Communication about the planning and implementation of the INTERSCALE project, motivation for the introduction of INTERCARE | Management team, project team | All employees in the LTCF | [preparatory month 2] | Through preparatory workshops and the INTERSCALE implementation handbook, provide a basis of knowledge and effectiveness of the INTERCARE model | Discuss the INTERSCALE implementation handbook.  Participate in preparatory workshops;  Present internal reasons for and opportunities offered by the INTERCARE model |
| Inform residents about the study with the information that non-participation be actively communicated | a) Adapt the information sheet to the context of the LTCF  b) Ensure that all residents are informed by means of an information sheet that they are actively informed about the study and have the opportunity to decline participation  c) Talking to residents who have questions about the study | Project team, management team  INTERCARE nurse | Management team, INTERCARE nurse | [preparatory month 2] | Provide information letters | Customize and distribute;  Record the number of opt-out requests and submit them to the study team |
| Information for physicians | Get physicians on board, clarify cooperation possibilities, which can be combined with a presentation of the INTERCARE nurse if necessary | Management team  INTERCARE nurse  internal physicians | All physicians, management team, INTERCARE nurse | As early as possible, send an information letter by [preparatory month 2] at the latest | Provide information letters and possibly presentations | Inform physicians; clarify personal readiness |
| Information for external partners | Provide information about the project to all external partners / other professions involved, e.g., Physio, Ergo, Logo, Pastoral care | Project team | Management team, project team  External partners | Until [baseline month] | Provide information letters and possibly presentations | Inform external partners and enter into discussions; clarify cooperation |
| CAS INTERCARE | Participation in CAS INTERCARE (depending on agreements between the study team and the management team) | INTERCARE nurse | 1-2 persons per institution | CAS participation, depending on the start date (ongoing) | Reserve study places | Registration for CAS;  Active participation;  Carry out self-tests;  Learn and practise training content;  Implement module completions in the LTCF |

| PROJECT TASK | DESCRIPTION | RESPONSIBLE PERSONS | PERSONS INVOLVED | TIME-  PERIOD / KEY DATE | Responsibilities | |
| --- | --- | --- | --- | --- | --- | --- |
|  |  |  |  |  | INTERSCALE study team | LTCF |
| Baseline month | | | | | | |
| Creation of a work plan for the INTERCARE nurse | a) Internal planning process for regular exchanges between INTERCARE nurse and individual units  b) Communicating the tasks of the INTERCARE nurse, in particular, the availability for coaching with the care teams | Unit leaders  Project team | Depending on the LTCF | [baseline month] | An overview of the possible tasks of the INTERCARE nurse is provided | Clarification of the scope of the INTERCARE nurse |
| Prepare implementation for each core element | Internal planning of implementation for each core element: people who should be involved, as well as tasks and responsibilities | Project team | Depending on the LTCF | [baseline month] | An overview of possible roles, respective tasks, and responsibilities is provided | Clarification of the areas of responsibility, specifically for those involved in the individual core elements |
| Structured form for reporting on costs and expenses in LTCFs related to implementation strategies (2b) | Data form via REDCap ®   - Recording of the implementation of the implementation strategies provided by the study team, including the time spent and wages of the persons involved - Recording of implementation strategies developed and implemented by the LTCFs, in addition to the strategies provided by the study team | People involved in the strategies | Depending on the LTCF | Months for cost documentation and deadline for transmission:  [1. quarter]  [2. quarter]  [3. quarter]  [4. quarter]  Monthly on the 15th of the following month | Provide REDCap® data form | Document expenses for strategies on an ongoing basis from [implementation phase month 1] |
| Questionnaire on the characteristics of the LTCF (2c_1) | Questionnaire to be completed via REDCap®  Topics:   - Size of the facility, number of units, staffing of the units, focus, medical model, etc. (T0) | One member of management completes one questionnaire per participating LTCF using LTCF data. | 1 person Management team | [baseline month] | Provide REDCap® questionnaire and a PDF version | Fill in the REDCap® data form; select who fills in according to criteria |
| Questionnaire on the characteristics of leadership (2c_2) | Questionnaire to be completed via REDCap®  Topics:   - Professional background, duration of employment in the LTCF (T0) - Acceptance and implementation of the INTERCARE model (T0 & T4) | The LTCF director, the director of nursing, and the project manager each complete one questionnaire per LTCF (if different people hold these roles). | Depending on LTCF | [baseline month] | Provide REDCap® questionnaire | Filling in the questionnaire, selecting who fills in according to criteria |
| Semi-structured interview to measure fidelity to the core elements of the INTERCARE model (2f) | Online interview with the study team  Topics:   - Status of implementation of the core elements (intervention fidelity, hindering & facilitating factors) | INTERCARE nurse and/or management team | INTERCARE nurse and/or management team | [baseline month] | Arrange an appointment, conduct and record the interview | The current status of implementation internally  Answer oral questions at the online appointment |
| INTERCARE nurse questionnaire (3b) | Online questionnaire via REDCap®  Topics:   - Professional background, duration of employment in the LTCF, and information on experience as an INTERCARE nurse - Quality of working environment, personnel outcomes - Perceived quality of care - Acceptance and feasibility of the INTERCARE model - Fidelity to interprofessional collaboration | INTERCARE nurse | All INTERCARE nurses | [baseline month] | Provide REDCap® questionnaire; define inclusion and exclusion criteria | Filling out the questionnaire |
| Staff questionnaire (4) | Online questionnaire via REDCap®  Topics:   - Professional background - Quality of working environment, personnel results - Perceived quality of care - Acceptance and feasibility of the INTERCARE model - Fidelity to evidence-based instruments and interprofessional collaboration | Staff who are in direct contact with residents in a nursing/care capacity. | All personnel who meet the criteria. | [baseline month] | Provide REDCap® questionnaire; define inclusion and exclusion criteria | Send a link to the questionnaire to all employees who meet the criteria; ask them to fill it out, and, if possible, make time available |
| Structured data export of routinely collected/administrative resident data (6a) | National quality indicators (pain, malnutrition, polymedication, physical restraint use) | RAI responsible person(s) or person(s) responsible via BESAQSys | Depending on LTCF | Data export for [baseline month]  Due date: xxx | Provision of the instructions for export/provision of the declaration of consent for data export by BESAQSys  Requesting LTCFs to submit data, checking the data received | RAI: Extract data and transmit to the study team  BESA: Give consent to BESAQSys for the export of data  Alternative: RAI and BESA export of data from the BESAQSys dashboard |
| Structured form for reporting on the fidelity of implementation of advance care planning (6c) | Filling out an Excel data form on the topics:  Implementation of the core element for new residents: Existence of the elements of medical emergency planning in consultation with residents and/or relatives in relation to life-prolonging measures/symptom relief (e.g., in relation to resuscitation, hospital admission, administration of antibiotics) | According to internal agreement: INTERCARE nurse, project management, administration, or other person | Depending on LTCF | One-off reporting for each new resident as soon as the first medical emergency order or treatment plan with basic questions for residents has been completed | Providing the Excel data form  Requesting LTCFs to submit data, checking the data received | Extract data and enter it in Excel |

| PROJECT TASK | DESCRIPTION | GROUP OF PERSONS CONCERNED | TIME PERIOD / KEY DATE | Responsibilities | |
| --- | --- | --- | --- | --- | --- |
|  |  |  |  | INTERSCALE study team | LTCFs |
| Implementation phase: [implementation months 1-12] | | | | | |
| Putting the model into practice | Each core element is implemented in the LTCFs based on the specially created project plan (if necessary, a work plan specific to each core element) | - INTERCARE nurse - Project team - All persons from the participating units | From [implementation month 1]  Time period for the introduction of ISBAR and STOP&WATCH: until [implementation month 10] | Support during implementation | Control and support by the project team and the project management on-site |
| Ongoing information for new employees about the INTERSCALE project | Inform employees about the implementation of the project | Management team or INTERSCALE project team | Ongoing throughout the project | Support during implementation | Use a suitable communication structure |
| Ongoing information for new residents | Residents / legal representatives are informed of the possibility to opt out of participation in the study upon admission to the institution | - INTERCARE nurse - Project management on site | Ongoing throughout the project | Provision of the information sheet | Informing residents/legal representatives about the project on admission |
| Ongoing completion and reflection of the reflection tool on hospital admissions | Collection of data throughout the entire project implementation phase;  Reflection is completed and discussed by the team under the direction of the INTERCARE nurse. | INTERCARE nurse | Ongoing | Provide a tool for reflection | Collecting the data (carrying out the reflection and completing the tool) and reflecting in the respective team involved |
| Concept for multidimensional geriatric assessment | Follow the steps in the action plan to frame the concept | INTERSCALE project team | [implementation months 1-8] | Provide samples  Support with challenges | Define concept |
| Coaching for INTERCARE nurses of the participating institutions | Coaching for role development | INTERCARE nurse | Continuously  (Information on exact arm-specific dates will be provided separately) | Organize and conduct coaching appointments (incl. preparation and follow-up) | Bring case studies;  Prepare and follow up coaching sessions;  Participate in coaching sessions |
| Exchange between the study team and the management team  and the INTERCARE nurse | Exchange on feasibility, local adaptations, challenges, development of a new team structure, quality monitoring, etc. | INTERCARE nurse / Management team | Every 2 months  (Information on exact arm-specific dates will be provided separately) | Organizing the meetings (mainly Zoom or Teams) | Participation in the meeting |
| Workshop on the core element of data-based quality development | Becoming familiar with the national quality indicators and the core element of data-based quality development | Management team, quality managers,  RAI and BESA managers | 1. quarter of the implementation period, date to be discussed | Organizing the workshop (Zoom or Teams) | Participation in the meeting |
| Structured data export of routine/administrative organizational data on personnel (1) | Sharing personnel resource planning (PEP) via SWITCHdrive | Depending on LTCF | Months for data export and cut-off date in the implementation phase:  [1. quarter]  [2. quarter]  [3. quarter]  [4. quarter]  Monthly on the 15th of the following month | Request LTCFs to transmit data; check data received | Perform internal data export |
| Structured form for reporting on the implementation of data-based quality improvement and multidimensional geriatric assessment (2a) | Degree of implementation fidelity for two core elements: Multidimensional geriatric assessment and data-based quality improvement | Depending on the internal responsibilities | [implementation phase month 12] | Inform the LTCFs which aspects are to be documented | Document in writing how the two core elements were implemented;  Send document to study team via Switchdrive |
| Structured form for reporting on operational costs and expenses related to implementation strategies (2b) | REDCap® questionnaire (online):   - Recording the implementation of the implementation strategies provided by the study team, including the time and salary of the people involved - Recording of implementation strategies developed and implemented by the LTCFs in addition to the strategies provided by the study team | People involved in the strategies | Months for cost documentation and deadline for submission in the implementation phase:  [1. quarter]  [2. quarter]  [3. quarter]  [4. quarter]  Monthly on the 15th of the following month | Provision of the REDCap® questionnaire | Document expenditure for strategies on an ongoing basis;  Submit monthly to the study team via REDCap® questionnaire |
| Questionnaire on the characteristics of leadership (2c_2) | Questionnaire to be completed via REDCap®  Topics:   - Upper management:   - Acceptance and feasibility of the INTERCARE model | The LTCF director, the director of nursing, and the project manager each complete one questionnaire per LTCF (if different people hold these roles). | [implementation phase month 12] | Provision of the REDCap® questionnaire | Fill in the questionnaire; select who fills in according to criteria |
| Questionnaire on prices, costs, and revenues of the LTCFs (2d) | Word or PDF document with details of   - Price list/tax regulations for residents - Information on the impact of hospital admissions of residents on the costs/income of the LTCF - Approx. wage of an INTERCARE nurse | Depending on LTCF | [implementation phase month 3] | Provide a document for data collection, remind of data transfer, and check the data | Compile documents, collect and forward data |
| Focus group on process evaluation of implementation strategies (2e) | Online focus group  Topics:   - Perception of how implementation strategies bring about change - Awareness of local contextual factors that influence implementation strategies | LTCF management, INTERCARE nurse, project managers, etc.: max. two persons per LTCF | [implementation phase month 12] | Arrange an appointment, conduct and record the interview | Discuss internal changes through the implementation strategies;  Answer questions orally at the online appointment |
| Semi-structured interview to measure fidelity (2f) | Online implementation with the study team  Topics:   - Status of implementation of the core elements (intervention fidelity, hindering & facilitating factors) | INTERCARE nurse and/or management team | [implementation phase month 3]  [implementation phase month 6]  [implementation phase month 9]  [implementation phase month 12] | Arrange an appointment, conduct and record the interview | Discuss the current status of implementation internally  Answer questions orally at the online appointment |
| Semi-structured interviews on experiences with the implementation strategies (a result of the process evaluation) (2g) | Experience with the implementation strategies developed by the study team | Depending on the LTCF: LTCF management/project manager / INTERCARE nurse | [implementation phase month 12] | Conducting interviews | Conducting interviews |
| Structured form for reporting on coaching activities (3a) | Upload a structured Word document via SWITCHdrive to:  Implementation of coaching measures for complex resident situations and situations with relatives with the care staff (documentation over three days in each case) | INTERCARE nurse | Trimonthly:  [implementation phase month 3]  [implementation phase month 6]  [implementation phase month 9]  [implementation phase month 12] | Providing the Word document | Document and upload via SWITCHdrive |
| INTERCARE nurse questionnaire (3b) | Online questionnaire via REDCap®  Topics:   - Quality of working environment, personnel outcomes - Perceived quality of care - Acceptance and feasibility of the INTERCARE model - Fidelity to interprofessional collaboration | INTERCARE nurse | [implementation phase month 6]  [implementation phase month 12] | Provision of the REDCap® questionnaire | Filling out the questionnaire |
| Participation in observation and informal survey to measure fidelity in relation to coaching and empowerment activities (3c) | Recording how the INTERCARE nurse performs various tasks (i.e., implementation of minimum requirements), organizes and structures coaching activities, and deals with acute situations | INTERCARE nurse | [implementation phase month 6] | Make an appointment, accompany the INTERCARE nurse for one day | Participation in observation and informal questioning |
| Staff questionnaire (4) | Online questionnaire via REDCap®  Topics:   - Professional background - Quality of working environment, personnel outcomes - Perceived quality of care - Acceptance and feasibility of the INTERCARE model - Fidelity to evidence-based instruments and interprofessional collaboration | Staff who are in direct contact with residents in a nursing/care capacity. | [implementation phase month 6]  [implementation phase month 12] | Provision of the REDCap® questionnaire | Completion of the questionnaire; selection of who completes according to criteria |
| Questionnaire physicians (5) | Online questionnaire via REDCap®  Topics:   - Professional background - Area of responsibility in the LTCF - Fidelity to the core elements | Physicians who care for residents in the LTCF (internal physicians, general practitioners, mobile physicians, part-time general practitioners) | [implementation phase month 6]  [implementation phase month 12] | Agree with the LTCF on which physicians should be included;  Distribute the questionnaire to all physicians | Submit the address list of physicians responsible in the LTCF to the study team |
| Structured data export of routinely collected/administrative resident data (6a) | National quality indicators (pain, malnutrition, polymedication, physical restraint use) | RAI responsible person(s) or person(s) responsible via BESAQSys | Months for data export and cut-off datein the implementation phase:  [1. quarter]  [2. quarter]  [3. quarter]  [4. quarter]  Monthly on the 15th of the following month | Provision of the instructions for export/provision of the declaration of consent for data export by BESAQSys  Requesting LTCFs to submit data; checking the data received | RAI: Extract and transmit data to the study team  BESA: Give consent to BESAQSys for the export of data |
| Structured data export on visits to emergency departments and hospital admissions of residents (6b) | Excel data form or internal systems, the format is agreed with LTCFs. Structured data export on visits to emergency departments and hospital admissions of residents | According to internal agreement: INTERCARE nurse, project management, administration, or other person | Months for data export and cut-off datein the implementation phase:  [1. quarter]  [2. quarter]  [3. quarter]  [4. quarter]  Monthly on the 15th of the following month | Provision of the Excel data form or consultation with the LTCF for a suitable format  Requesting LTCFs to submit data, checking the data received | Extract data and enter it into Excel or a selected format |
| Structured form for reporting on the fidelity of implementation of advance care planning (6c) | Filling out an Excel data form on the topics:  Implementation of the core element for new residents: Existence of the elements of medical emergency planning in consultation with residents and/or relatives in relation to life-prolonging measures/symptom relief (e.g., in relation to resuscitation, hospitalization, administration of antibiotics) | According to internal agreement: INTERCARE nurse, project management, administration, or other person | One-off reporting for each new resident as soon as the first medical emergency order or treatment plan with basic questions for residents has been completed | Providing the Excel data form  Requesting LTCFs to submit data; checking the data received | Extract data and enter it in Excel |

| PROJECT TASK | DESCRIPTION | RESPONSIBLE PERSONS | TIME PERIOD | Responsibilities | |
| --- | --- | --- | --- | --- | --- |
|  |  |  |  | INTERSCALE study team | LTCFs |
| Sustainment phase: [sustainment phase months 13-24] | | | | | |
| Maintain structures and processes that were introduced in the implementation phase for the individual core elements | The persons involved continue to carry out their tasks and responsibilities in connection with the care model | Depending on the core element and the structures in the LTCF | Continuous | / | Ensure that structures and processes are continued |
| Ongoing information for new employees about the INTERCARE project | Inform employees about the implementation of the project | Management team or INTERSCALE project team | Ongoing throughout the project | / | Use a suitable communication structure |
| Ongoing information for new residents | Residents / legal representatives are informed of the possibility to opt out of participation in the study upon admission to the institution | - INTERCARE nurse - Project management on-site | Ongoing throughout the project | / | Informing residents/legal representatives about the project on admission |
| Structured data export of routine/administrative organizational data on personnel (1) | Sharing personnel resource planning (PEP) via SWITCHdrive | Depending on LTCF | Months for data export and cut-off date in the sustainment phase:  [5. quarter]  [6. quarter]  [7. quarter]  [8. quarter]  Monthly on the 15th of the following month | Requesting LTCFs to submit data; checking the data received | Perform internal data export |
| Structured form for reporting on operational costs and expenses related to implementation strategies (2b) | REDCap® questionnaire (online):   - Recording of the implementation of the implementation strategies provided by the study team, including the time spent and wages of the persons involved - Recording of implementation strategies developed and implemented by the LTCFs in addition to the strategies provided by the study team | People involved in the strategies | Months for cost documentation and deadline for submission in the sustainment phase:  [5. quarter]  [6. quarter]  [7. quarter]  [8. quarter]  Monthly on the 15th of the following month | Provide REDCap® data form | Continuously document effort for strategies and forward to the study team |
| Questionnaire on prices, costs, and revenues of the LTCFs (2d) | Word or pdf document with details of   - Price list/tax regulations for residents - Information on the impact of hospital admissions of residents on the costs/income of the LTCF - Approx. wage of the INTERCARE nurse | Administration, management team | [Sustainment phase month 15]  [Sustainment phase month 24] | Provide a document for data collection, remind of data transfer, and check the data | Compile documents, collect and forward data |
| Focus group on process evaluation of implementation strategies (2e) | Online group survey  Topics:   - Perception of how implementation strategies bring about change - Awareness of local contextual factors that influence implementation strategies | Management team, INTERCARE nurse, project managers, etc.: max. two persons per LTCF | Sustainment phase month 21] | Arrange an appointment, conduct and record the interview | Discuss internal changes through the implementation strategies;  Answer questions orally at the online appointment |
| Semi-structured interview to measure fidelity (2f) | Online implementation with the study team  Topics:   - Status of implementation of the core elements (intervention fidelity, hindering & facilitating factors) | INTERCARE nurse and/or management team | Trimonthly:  [Sustainment phase month 15]  Sustainment phase month 18]  Sustainment phase month 21]  [Sustainment phase month 24] | Arrange an appointment, conduct and record the interview | Discuss the current status of implementation internally  Answer questions orally at the online appointment |
| Semi-structured interviews on experiences with the implementation strategies (a result of the process evaluation) (2g) | Experience with the implementation strategies developed by the study team | Depending on the LTCF: LTCF management/project manager / INTERCARE nurse | [Sustainment phase month 24] | Conducting interviews | Conducting interviews |
| Structured form for reporting on coaching activities (3a) | Upload a structured Word document via SWITCHdrive to:  Implementation of coaching measures for complex resident situations and situations with relatives with the nursing and care staff (documentation over three days in each case) | INTERCARE nurse | Trimonthly:  [Sustainment phase month 15]  Sustainment phase month 18]  Sustainment phase month 21]  [Sustainment phase month 24] | Providing the Word document | Document and upload via SWITCHdrive |
| Structured data export of routinely collected/administrative resident data (6a) | National quality indicators (pain, malnutrition, polymedication, physical restraint use) | RAI responsible person(s) or person(s) responsible via BESAQSys | Months for data export and cut-off date in the sustainment phase:  [5. quarter]  [6. quarter]  [7. quarter]  [8. quarter]  Monthly on the 15th of the following month | Provision of the instructions for export/provision of the declaration of consent for data export by BESAQSys  Requesting LTCFs to submit data; checking the data received | RAI: Extract and transmit data to the study team  BESA: Give consent to BESAQSys for the export of data |
| Structured data export on visits to emergency departments and hospital admissions of residents (6b) | Excel data form or internal systems, the format is agreed upon with the LTCFs:  Structured data export on visits to emergency departments and hospital admissions of residents | According to internal agreement: INTERCARE nurse, project management, administration, or other person | Months for data export and cut-off date in the sustainment phase:  [5. quarter]  [6. quarter]  [7. quarter]  [8. quarter]  Monthly on the 15th of the following month | Provision of the Excel data form or consultation with the LTCF for a suitable format  Requesting LTCFs to submit data, and checking the data received | Extract data and enter it into Excel or a selected format |
| Structured form for reporting on the fidelity of implementation of advance care planning (6c) | Filling out an Excel data form on the topics:  Implementation of the core element for new residents: Existence of the elements of medical emergency planning in consultation with residents and/or relatives in relation to life-prolonging measures/symptom relief (e.g. in relation to resuscitation, hospitalization, administration of antibiotics) | According to internal agreement: INTERCARE nurse, project management, administration, or other person | One-off reporting for each new resident as soon as the first medical emergency order or treatment plan with basic questions for residents has been completed | Provision of the Excel data form;  Requesting LTCFs to submit data, checking the data received | Extract data and enter in Excel |
