# Supplementary material for: Sustainable improvement of interprofessional care for better resident outcomes: protocol for the INTERSCALE hybrid type III effectiveness cluster-randomized trial comparing individualized and collaborative delivery of an evidence-based care model for long-term care
Source: Implement Sci. 2026 Feb 20;21:24. doi: 10.1186/s13012-026-01489-0 (PMC13032367; doi:10.1186/s13012-026-01489-0)
Supplement: Supplementary file 5 — Supplementary Material 5. [file 13012_2026_1489_MOESM5_ESM.pdf]

Declaration of consent for observation and participation in an informal interview - INTERCARE nurse

Director of Studies: PI

Project manager: lead of sub-study

Project coordinator: xxx

Dear Ms. ....
Dear Mr. ....

You have been appointed as an INTERCARE nurse at [*name of institution*] as part of the INTERSCALE research project. You are an essential interface in the INTERCARE model. We are pleased that you are involved in the project and actively support it with your work. As part of the project evaluation, we would like to observe you at work for a day and conduct an informal interview to record how the implementation of the care model is progressing in general, and your role in particular.

The guidelines for good ethical research stipulate that participants in a research project must explicitly and comprehensibly agree to participate in the project by signing and attesting that they are doing so voluntarily.

For this reason, we would like to ask you to read and sign this declaration of consent.

You have already received information about the INTERSCALE project. We have listed the most important information regarding the observation and participation in the informal interviews below.

What do we want to achieve with the research project?

As part of the INTERSCALE project, we are introducing a nurse-led care model in 40 Swiss long-term care facilities (LTCFs) to improve care quality, strengthen interprofessional collaboration, and reduce unplanned hospital admissions. The introduction of the INTERCARE nurse role is the most essential core element of this care model.

In this project phase, we would like to examine the implementation of the core elements in LTCFs. To gain comprehensive insights from the people involved, we will therefore conduct an observation and an informal discussion with you, an INTERCARE nurse.

What does participating in the observation and interview mean to you

We will observe you over the course of a day as you work in the LTCF and seek to talk to you. We will take notes as we do so. The observation period lasts up to six hours. At the beginning of the observation, we will once again explain the purpose of the observation and the informal discussion. Topics in the interview and observation will include activities to support and coach care teams, the assessment of residents in acute situations, and communication within the interprofessional team. At the end of the observation, we will conduct an informal interview with you lasting approximately half an hour to reflect on the situations from the observation sequence and to determine to what extent they were typical of your work as an INTERCARE nurse. If you agree, we will record the interview digitally.

What benefits and risks are associated with the observation and participation in the informal interview for you?

By being available for observation and informal interviews, you will help us gain important insights into the implementation of the core elements. Participation has no direct benefit for you and is not associated with any risks.

What rights do you have if you take part in the observation and informal interview?

Participation in the interviews and observation is voluntary. You can withdraw your participation at any time. You do not have to justify your decision, and you will not suffer any disadvantages. If you withdraw from the observation, the interview, or the entire project prematurely, we will continue to use the data collected from you to describe the implementation of the model in your LTCF. You may ask questions about your participation, your role, and the project at any time.

What happens to your data?

We comply with all legal data protection regulations. Your data will only be used within the framework of the research project. Only members of the research group have access to it. The data will be kept in written notes, possibly an audio file, and a transcription. The data will be stored in such a way that all identifying elements are removed, and it is not possible to identify you or the LTCF in which the observation took place. All persons involved in the research project are bound to confidentiality. The collected data will be used to produce a national, freely accessible report on the outcomes of the care model. On the other hand, further publications will be made in national and international journals. We will handle all publications so that it will not be possible to identify you. If we wish to use a personal quotation from you by name, we will always clarify this with you first and will only do so with your written consent.

Do you have any questions?

If you have any questions about the observations or interviews, please contact the project coordinator. If you have any questions that the project coordinator cannot answer, please get in touch with the study director.

The original of this consent form will be stored securely by the INTERSCALE research team. You will receive a copy.

Declaration of consent:

I hereby confirm that I have read and fully understood all of the above information and agree to participate in the observation and informal interview.

Place, date	Signature of participating INTERCARE nurse

Place, date	Signature of project manager (lead of sub-study)
