# Supplementary material for: Sustainable improvement of interprofessional care for better resident outcomes: protocol for the INTERSCALE hybrid type III effectiveness cluster-randomized trial comparing individualized and collaborative delivery of an evidence-based care model for long-term care
Source: Implement Sci. 2026 Feb 20;21:24. doi: 10.1186/s13012-026-01489-0 (PMC13032367; doi:10.1186/s13012-026-01489-0)
Supplement: Supplementary file 6 — Supplementary Material 6. [file 13012_2026_1489_MOESM6_ESM.pdf]

Information for residents on the INTERSCALE study

Dear Ms.   / Dear Mr.,

We would like to inform you about a study in which our long-term care facility (LTCF) is participating.

As part of the INTERSCALE study, our LTCF will introduce the "INTERCARE" nurse-led care model. The study is being conducted by the *University*.

It is important to us to offer good treatment and care for you as a resident. We are therefore introducing a new model. Registered nurses (INTERCARE nurses) will receive in-depth training to support care teams in acute or complex resident situations. They support the teams in assessing health situations. They promote the exchange of information with relatives and physicians to implement appropriate measures. Studies show that such models improve the quality of care. The INTERSCALE study examines which measures can support homes when introducing a model such as INTERCARE.

The INTERCARE model

INTERCARE is a nurse-led model that was developed in Switzerland and successfully implemented in 11 LTCFs between 2018 and 2020. The core elements include

- Introduction of a geriatric specialist nurse
- Strengthening interprofessional collaboration
- Introduction of instruments to strengthen communication within the team
- Implementation of advance care planning
- Data-based quality improvement

Initial results show that the INTERCARE model helps identify changes in residents' health situations earlier. In addition, residents' wishes regarding their treatment can be well taken into account, and unplanned hospital admissions, which are often stressful for residents, can be reduced.

The INTERSCALE study

The INTERSCALE study is investigating how best to support LTCFs when they introduce the INTERCARE model. Our LTCF will participate from *Month Year* to *Month Year*. During this time, we will collect various data from you and submit it to the study team. This includes:

- **Advance care planning:** For all residents entering the home from *Month Year*, we document when you first came to the LTCF (date of entry) and when we spoke to you about the following topics
  - Wishes for resuscitation
  - Place of treatment in the event of deterioration in health
  - Antibiotic treatment

  We only inform the study team whether a corresponding interview has taken place. We do not transmit the content of conversations.

- **Hospital admissions:** We make a note of every transfer to the hospital, whether with or without hospital admission:
  - Date of the transfer
  - whether the transfer was planned or unplanned
  - Reason for transfer (e.g., fall, shortness of breath)
  - Place of transfer (acute hospital, rehabilitation, psychiatric ward)
  - Date of release

- **National quality indicators:** We submit fully anonymized data on pain, weight loss, physical restraint use, and polypharmacy. This data comes from the needs assessment we conduct regularly.

All data is transmitted to the study team in such a way that no one can draw conclusions about you personally. The LTCF will receive regular graphical evaluations of these topics from the study team to monitor changes over time and compare results with other LTCFs. The study team will analyze and publish the data scientifically. It will not be possible to draw conclusions about participating LTCFs or residents. The data will be stored securely at the *University* and deleted at the end of the study.

Between 2024 and 2027, 40 homes will take part in the study. The aim is to promote the long-term implementation of new models and support LTCFs in developing the quality of care.

Your Participation

The study has been classified as a quality assurance project by the Ethics Committee of Northwestern and Central Switzerland (EKNZ). You are not required to give informed consent to participate in the study. However, you have the option to refuse participation. If you do not agree to the collection and transmission of data to the study team, please let us know in person, by phone, or in writing by <Month Year>. Without your feedback, we will include you in the study.

You will find the most important information again in the enclosed flyer. Detailed information on the INTERCARE model and the INTERSCALE study can be found on the study website: *URL*. Here you will also find information on published articles or reports.

If you have any questions, please do not hesitate to contact us:

Local contact person:

Telephone number:

E-mail address:

If you have any further questions, please get in touch with the study team at the *University*

Responsible: xxx

Project coordinator: xxx

E-mail address: xxxmailto:nip@unibas.ch
